# Evaluating the impact of cannabinoid extracts on chronic pain and quality of life in endometriosis patients – a pilot study

**DOI:** 10.1371/journal.pone.0356768

**Published:** 2026-09-22

**Authors:** Janosch W. Kratz, Renata Voltolini Velho, Sandra Bock, Jalid Sehouli, Sylvia Mechsner

**Affiliations:** Department of Gynecology Charité with Centre of Oncological Surgery, Endometriosis Research Centre Charité, Berlin, Germany; Noorda College of Osteopathic Medicine, UNITED STATES OF AMERICA

## Abstract

Endometriosis is a chronic gynecological disorder characterized by persistent pain and impaired quality of life, with limited effective treatment options. Emerging evidence indicates that cannabinoids may provide analgesic and symptom-relieving effects in chronic pain conditions. This prospective single-center cohort study investigated the effects of standardized oral cannabis extracts (cannabinoid-based medicinal products, CBMPs) on pain and quality of life in women with endometriosis. Thirty premenopausal women aged 18–45 years with confirmed endometriosis and chronic pelvic pain were enrolled; 27 completed the 3-month treatment period. Participants received oral CBMPs containing a balanced THC: CBD ratio. Pain intensity, frequency of pain episodes, analgesic use, fatigue, anxiety, central sensitization, disability, and quality of life were assessed using validated questionnaires (VAS, EHP-30, PDI, CFS, GAD-7, CSI). Significant improvements were observed after one month of treatment, with continued benefit over three months. Pain intensity decreased markedly (p = 0.0004), accompanied by reductions in the number of pain days and analgesic use, including both NSAIDs (e.g., ibuprofen, diclofenac) and opioid analgesics (e.g., tramadol, tilidin, oxycodone) (p < 0.001). Fatigue (p = 0.0013), anxiety (p < 0.0001), central sensitization (p < 0.0001), disability (p < 0.0001), and overall quality of life (EHP-30) improved significantly. Treatment was well tolerated; side effects were predominantly mild (severity score 1 on a 0–3 scale) and transient, occurring mainly during the titration phase and declining thereafter. Although limited by its single-center design, lack of placebo control, and short follow-up, this study provides preliminary evidence that oral CBMPs may represent a safe and effective adjunct therapy for managing pain and related symptoms in women with endometriosis. Larger randomized controlled trials are warranted to confirm these findings and establish optimal dosing strategies.

## Introduction

Endometriosis is a chronic, benign, yet debilitating gynecological disorder characterized by ectopic endometrial-like tissue, primarily within the pelvic cavity. This estrogen-dependent inflammatory condition causes severe dysmenorrhea, dyspareunia, chronic pelvic pain, and infertility, significantly impairing quality of life and reproductive health [1,2]. Pain in endometriosis arises not only from inflammation but also from neurogenic sensitization, reflecting the complex pathophysiology underlying symptom persistence [3,4]. Globally, approximately 10% of reproductive-age women are affected, but non-specific symptoms and diagnostic challenges often delay treatment [2,5]. Current treatments, including hormonal therapies and laparoscopic surgery, aim to suppress lesion growth and alleviate symptoms but often provide limited or temporary relief [1,6,7]. Pharmacological treatments, including hormonal agents and analgesics, may be poorly tolerated due to side effects, emphasizing the need for alternative therapies [8]. Nonsteroidal Anti-Inflammatory Drugs (NSAIDs), including ibuprofen and diclofenac, are the standard first-line pharmacological treatment for endometriosis-related pain. Despite this widespread use, prolonged NSAID therapy carries gastrointestinal, renal, and cardiovascular risks. Furthermore, many patients continue to experience inadequate symptom control even with consistent NSAID use [9]. In cases where NSAIDs are ineffective, opioid analgesics are often prescribed. This practice raises significant concerns about dependence, tolerance, and systemic side effects, which further increase the disease burden [10]. As a result, the increasing reliance on opioids in chronic pelvic pain management highlights the urgent need for adjunct or alternative therapies that can reduce overall pharmacological exposure while maintaining or improving pain control [1,11].Given these limitations, many women adopt self-management strategies to relieve symptoms and enhance quality of life [11–14]. These include physical activity (yoga, Pilates), lifestyle modifications (diet, rest), and complementary methods such as acupuncture, herbal medicine, and cannabis. In a recent survey of 912 endometriosis patients, 345 reported using cannabinoids for symptom management: 231 used CBD or hemp oil, and 114 used THC-containing cannabis flowers (source unspecified) [11]. Cannabis was rated the most effective symptom relief method (mean score 7.6/10), with ~90% of users also reducing conventional pain medication [14]. While promising, these outcomes require confirmation in rigorously designed clinical studies.

Cannabinoids are increasingly investigated as adjunct therapies for chronic pain, including endometriosis-associated pain. Δ9-Tetrahydrocannabinol (THC) and cannabidiol (CBD) modulate the endocannabinoid system (ECS) to produce analgesic, anti-inflammatory, antispasmodic, anxiolytic, and mood-stabilizing effects [15,16]. As the ECS regulates pain, inflammation, and immune responses, and is thought to be dysregulated in endometriosis, it presents a promising therapeutic target [15,17–19].

Preclinical studies indicate that cannabinoids may inhibit lesion proliferation, modulate immune responses, and reduce neuroinflammation and nociceptive signaling [19–23]. However, clinical evidence, particularly regarding the real-world effectiveness, remains limited [24].

This observational study aimed to evaluate the effects of oral cannabis extracts containing standardized concentrations of THC and CBD on chronic pelvic pain, symptom burden, and quality of life in patients with confirmed endometriosis. By capturing patient-reported outcomes over a three-month treatment period, this study seeks to provide preliminary data on the therapeutic potential and tolerability of oral cannabinoid-based medicinal products (CBMPs) in this complex patient population, complementing existing clinical research and informing future randomized controlled trials.

## Materials and methods

### Study design and study population

This is a prospective, single-center cohort study conducted at the Endometriosis Center Charité in Berlin, Germany. Patients with a confirmed diagnosis of endometriosis and pain resistant to both hormonal and analgesic treatment were invited to participate. After receiving both written and verbal information, all participants provided written informed consent before being included in the study. Data were collected exclusively through online questionnaires completed at baseline (before treatment started) and after 1, 2, and 3 months of treatment. No additional interventions were carried out as part of the study; all concomitant medications were continued without changes, and information on medication use was obtained via questionnaires. The primary outcome was a reduction of 15 points in the pain domain of the Endometriosis Health Profile (EHP-30) after three months of treatment. Secondary outcomes included changes in pain intensity, frequency of pain episodes, side effects, and multiple accompanying symptoms, assessed with validated questionnaires such as the Pain Disability Index (PDI), Chalder Fatigue Scale (CFS), Generalized Anxiety Disorder (GAD-7), and Central Sensitization Inventory (CSI).

Patients aged 18 to less than 49 years with a confirmed diagnosis of endometriosis—based on medical records, surgical reports, and/or histology—and who are premenopausal were eligible for participation. Additional inclusion criteria included experiencing chronic acyclic pelvic pain for at least 3 months with a mean pain intensity score of 4 or higher out of 10 on a visual analog scale (VAS), along with an independent clinical indication for treatment with CBMPs extracts as documented by a prescribing physician at Charité. Exclusion criteria consisted of: (i) cardiovascular disease (such as pacemaker use, myocardial damage, severe arrhythmias, or other serious cardiac conditions), (ii) pregnancy, breastfeeding, or plans to become pregnant, (iii) regular use of cannabinoids (either for medical or recreational purposes) within the past six months, (iv) starting or changing hormonal therapy within the three months before study entry or during the study, (v) participation in other clinical trials concurrently, (vi) known addiction disorders, and (vii) frequent operation of vehicles, buses, or heavy machinery.

Eligible patients were screened by a study physician who reviewed the medical documentation, medication plan, and inclusion/exclusion criteria. Patients fulfilling all criteria were formally enrolled after providing informed consent. Participants were informed that withdrawal from the study was possible at any time without providing reasons.

Between November 1, 2023 and May 31, 2025, 30 women were enrolled. One participant discontinued during the initial titration phase due to side effects, and two participants were lost to follow-up due to missing questionnaires despite repeated reminders. Thus, the final study population comprised 27 participants. The sample size of 30 was pragmatically defined based on recruitment feasibility within a single-center setting and is consistent with comparable early-phase observational pilot studies in this field. This study should therefore be interpreted as an exploratory pilot study, with the primary aim of evaluating feasibility, tolerability, and preliminary efficacy, rather than providing definitive estimates of treatment effect.

The study was approved by the Institutional Review Board of the Charité - University Medical Centre (Ethics approval number EA4/140/23). All patients provided written informed consent.

### Intervention

All participants received oral CBMPs (chemotype 2, balanced THC:CBD ratio) as an add-on therapy to their ongoing treatment. The dosing regimen at the Charité Endometriosis Center involved a slow, gradual titration over four weeks to minimize adverse effects and optimize tolerability. After the titration phase, the target daily dose was approximately 10 mg THC and 12 mg CBD (from an extract containing 12 mg THC and 14 mg CBD per ml), divided into two doses: 0.3 ml (3.6 mg THC / 4.2 mg CBD) in the morning and 0.5 ml (6.0 mg THC / 7.0 mg CBD) in the evening. To monitor safety and allow for individualized dose adjustments, participants were contacted by telephone after two and four weeks of treatment. These consultations aimed to identify side effects early and to optimize dosing if necessary. No additional interventions or changes to concomitant medications were made as part of the study. Adverse events were assessed using a structured self-report questionnaire at each study time point. Predefined items included nausea, fatigue, concentration difficulties, and mood-related symptoms. Other symptoms were not systematically pre-specified but could be reported in free-text fields.

### Statistical analysis

Statistical analyses were conducted using GraphPad Prism version 8.4.2 and IBM SPSS for Windows version 29.0.0.0. Data distributions were assessed using appropriate normality tests to determine whether parametric or nonparametric methods should be employed. Descriptive statistics are presented as mean ± standard deviation (SD), unless indicated otherwise. Comparisons between two related samples were performed using the paired t-test or the Wilcoxon signed-rank test, depending on data distribution. For comparisons involving more than two related groups, the Friedman test followed by Dunn’s post hoc correction for multiple comparisons was applied. Statistical significance was set at *p < 0.05, **p < 0.01, ***p < 0.001, and ****p < 0.0001.

## Results

### Baseline characteristics of the study population

This prospective, single-center cohort study included 27 women with confirmed endometriosis (Table 1). Participants had a mean age of 32.9 ± 5.1 years and a mean age at menarche of 12.2 ± 1.8 years. Nine women (33.3%) reported a history of pregnancy. The majority of patients (81.5%) were receiving hormone therapy, and 59.3% were bleeding-free at baseline. Among the prescribed regimens, Relugolix-CT was most frequent (45.5%), which, together with GnRH agonists, accounted for 59.1% of cases. Progestin-only therapies, such as Drospirenone and Dienogest, as well as local hormonal treatment with a levonorgestrel (52 mg) releasing device, comprised 22.7%. Combined estrogen-progestin oral contraception accounted for 18.2% of prescriptions. An important inclusion criterion was that no hormonal therapy could be started or changed within three months before study entry or during the study period, ensuring stability of background treatment throughout. CBMPs were administered strictly as add-on therapy to ongoing regimens. A known family history of endometriosis was reported in 18.5% of participants.

**Table 1:** Baseline characteristics of the study population.

	Mean/number	SD/percentage
**Age (years)**	**32.92**	**5.13 SD**
**Age at first menstrual period**	**12.16**	**1,77 SD**
**Pregnancy (number of patients)**		
Yes	9	33.33
No	18	66.67
Miscarriages	2	7.41
Abortion	4	14.81
**Surgeries**	**1.93**	**1.33 SD**
1	14	51.85
2	8	29.63
3	1	3.70
4	1	3.70
More than 4	3	11.11
**rASRM Stage**		**%**
I	14	51.85
II	5	18.52
III	5	18.52
IV	3	11.11
**Hormone Therapy**		**%**
Yes	22	81.48
No	5	18.52
**Bleeding free**		**%**
Yes	16	59.26
No	11	40.74
**Other family cases**		%
Yes	5	18.52
No	17	62.96
Unknown	5	18.52
**Pain intensity in the last month**	**6.81**	**1.27**
**Days/month taking painkillers**	**24.3/ 30**	**8.76/ 25-30**
**Days in pain despite the use of painkillers**	**14.93/ 13**	**10.88/ 5-30**
**Cannabis consume**		**%**
Never	10	37.04
Tried many years ago	12	44.44
A few times a year	4	14.81
Uses occasionally	1	3.70
Consumes regularly (at least 2x/week)	0	0
**Consume to relieve EM-associated symptoms**		
Yes	4	14.81
No	23	85.19

In terms of disease severity, 51.9% were classified as rASRM stage I, 18.5% as stage II, 18.5% as stage III, and 11.1% as stage IV. The mean number of prior surgeries was 1.9 ± 1.3, with 51.9% having undergone one surgery, 29.6% having undergone two surgeries, and 18.5% having undergone three or more surgeries.

Pain assessment during the month before treatment revealed a mean intensity of 6.8 ± 1.3 on a 0–10 VAS. Patients reported using analgesics on a mean of 24.3 ± 8.8 days per month (median 30, interquartile range [IQR] 25–30), with persistent pain on 14.9 ± 10.9 days (median 13, IQR 5–30) despite medication. Patients reported extensive exposure to conventional pharmacological pain management. Nearly all patients had used non-steroidal anti-inflammatory drugs (NSAIDs) such as ibuprofen or diclofenac, which represent the standard first-line therapy for endometriosis-related pain. However, despite frequent use, these drugs were insufficient to control symptoms, with many patients still reporting high baseline pain scores. In addition, the cohort demonstrated substantial use of paracetamol and metamizol, while a subset had experience with opioid medications such as tramadol, tilidin, or oxycodone. At the time of baseline assessment, seven participants were actively taking opioids, highlighting the refractory nature of their pain. Notably, 12 patients had never used opioids, underscoring the heterogeneity of pharmacological exposure within the group.

In parallel to pharmacological strategies, most patients had explored complementary and lifestyle interventions. The most frequently reported were dietary modifications (88.9%), yoga (88.9%), osteopathy (85.2%), and physiotherapy (74.1%). While acupuncture and psychotherapy were less commonly used, a considerable number of participants engaged in pelvic floor training or rehabilitation programs. Importantly, patients reported that interventions such as dietary changes, physiotherapy, and structured rehabilitation yielded the highest rates of meaningful improvement, suggesting that non-pharmacological strategies may play an important role in comprehensive care.

Cannabis use (either prescribed by a physician or acquired on the black market) at baseline was generally low. 37.0% had never used cannabis, 44.4% had tried it only in the distant past, and 14.8% reported using it specifically to relieve endometriosis-related symptoms. No participants reported regular use (≥2 times/week). The majority of patients (85.2%) had never used cannabis for endometriosis-associated symptom relief, and no participants reported regular use (≥2 times/week).

### Treatment Outcomes with oral CBMPs

#### Dosage and tolerability.

All participants received oral CBMPs (chemotype 2, balanced THC:CBD ratio with 12mgTHC/14mgCBD per ml) as an add-on therapy to their ongoing treatment. After a slow and gradual titration over 4 weeks, the target daily dose was approximately 10 mg THC and 12 mg CBD, divided into two doses: 0.3 ml (3.6 mg/4.2 mg of THC/CBD) in the morning and 0.5 ml (6 mg/7.0 mg of THC/CBD) in the evening. Throughout the 3-month treatment period, cannabis extract dosage remained stable, with median daily doses of 0.3 ml (IQR 0.3–0.3) in the morning and 0.5 ml (IQR 0.4–0.5) in the evening. The lack of dose escalation suggests that therapeutic effects were achieved at relatively low and steady levels, and that tolerance did not develop over the course of treatment. Fatigue, concentration, and memory complaints were the most persistent side effects, affecting around 60% of participants throughout. Most other symptoms decreased over time, e.g., anxiety/panic (26% → 4%) and paranoia (4% → 0%). Nausea was reported by a minority of participants. It also decreased: 29.6% at month 1, 25.9% at month 2, and 14.8% at month 3. One participant reported diarrhea at month 3. No vomiting or abdominal discomfort was reported. Some participants reported changes in appetite across the treatment period. Specifically, 44.4% reported changes at month 1, 55.6% at month 2, and 40.7% at month 3. Median severity scores were consistently low at all time points. This suggests that although appetite changes were relatively common, their intensity was mild and did not cause clinically significant adverse effects. Body weight was not systematically recorded in this study. As a result, formal assessment of weight change remains a priority for future investigations. Regarding cardiovascular symptoms, 18.5% of participants reported increased heart rate at months 1 and 2. This rate declined to 11.1% at month 3, with a median severity score of 0 at all time points. This indicates that, when present, it was not perceived as distressing or functionally limiting. “Mild” refers to a severity score of 1 on the 0–3 scale (noticeable but not distressing or functionally limiting). “Transient” refers to effects that were most prevalent during the titration phase and declined substantially thereafter. Severity scores were generally low, with most side effects having median values of 0–1 across all time points (Supplemental Material S1 File).

When the severity of side effects was expressed as the mean score per item (0–3), cannabinoid dose was weakly and negatively correlated with severity (Spearman’s r = −0.24, p = 0.03). When severity was expressed as the sum of all side effect scores, dose showed a moderate positive correlation with severity (r = 0.49, p < 0.001). The difference in direction (negative vs. positive) comes from the scaling. The mean index is bounded (0–3) and may mask increases when patients report many mild effects, while the sum index better reflects overall burden and seems to show a clearer dose–response.

As additional analysis, the dose groups (low, median, high) were created by tertiles of the total daily dose (sum of morning and evening). Nearly all participants reported at least one side effect regardless of dose group (Low: 100%, 10/10; Medium: 97%, 35/36; High: 97%, 34/35). Fisher’s exact test indicated no difference between groups (p = 1.00). Given the high prevalence, the analysis was not able to detect any dose–response relationship.

### Pain outcomes and reduction in analgesic medication use

**Fig 1:**
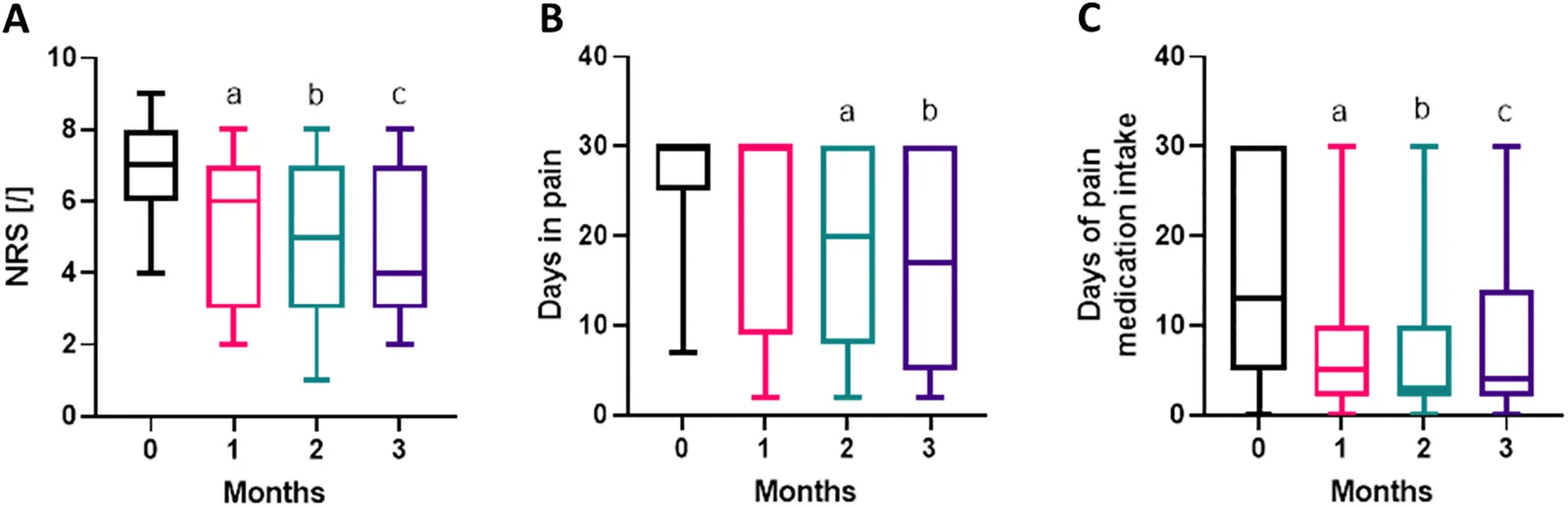
Clinical effects of oral CBMPs on pain and analgesic consumption in endometriosis (Friedman test). **(A) Pain intensity scores** (0–10 scale) at baseline (0 month) and after 1, 2, and 3 months of treatment. Patients showed a significant reduction in pain over time (overall p < 0.0001). Pairwise comparisons vs. baseline: a = p = 0.0020; b = p < 0.0001; c = p = 0.0004. **(B) Number of pain days per month** at baseline and during treatment. The frequency of pain episodes decreased significantly (overall p < 0.0001). Pairwise comparisons vs. baseline: a = p = 0.0003; b = p = 0.0005. **(C) Number of days requiring analgesic medication** before and during treatment. Cannabis extract significantly reduced analgesic use (overall p = 0.0009). Pairwise comparisons vs. baseline: a = p = 0.0266; b = p = 0.0094; c = p = 0.0266.

The use of oral CBMPs resulted in a clinically and statistically significant reduction in pain intensity (Fig 1A; p < 0.0001). Compared with baseline (6.81 ± 1.27), pain scores decreased as early as the first month (5.15 ± 1.89; p = 0.0020), with sustained and even greater improvements observed at months 2 (4.59 ± 2.08; p < 0.0001) and 3 (4.93 ± 1.94; p = 0.0004). The reduction in pain days was similarly notable (baseline median 30, IQR 25–30), with significant improvements after 2 (Fig 1B; median 20, IQR 8–30; p = 0.0003) and 3 months (median 17, IQR 5–30; p = 0.0005). These findings are particularly relevant given the high proportion of participants who reported persistent pain despite frequent analgesic use at baseline. CBMPs not only reduced the intensity of pain but also the frequency and chronicity of painful episodes, both of which are critical contributors to quality of life in endometriosis.

The number of days requiring analgesics significantly decreased (Fig 1C; p = 0.0009), with measurable improvements already at 1 month (p = 0.0266). This suggests that oral CBMPs may serve as an opioid-sparing and NSAID-sparing agent, reducing the dependence on medications associated with limited effectiveness and/or adverse effects. Seven participants were actively taking opioids at baseline, and the significant reduction in total analgesic days therefore represents a combined NSAID- and opioid-sparing effect, which is particularly clinically relevant given the well-known risks of long-term opioid use in chronic pain conditions. The reduction in pharmacological burden may carry health-economic implications; however, any net benefit must be interpreted cautiously, as the cost of CBMPs varies considerably across health systems and is not uniformly covered by public insurance. A formal pharmacoeconomic evaluation weighing CBMP costs against reductions in conventional analgesic use and associated adverse events is required before definitive conclusions can be drawn.

It is important to note that, in this cohort, pain intensity did not differ between women with and without hormone treatment (median 7, IQR 6–8 vs 7, IQR 6–7; p = 0.63, r = –0.14). However, hormone users reported substantially more pain days per month (30, IQR 30–30 vs 8, IQR 7–30; p = 0.07, r = –0.45). Given the small sample size and imbalance between groups, these findings should be interpreted cautiously. Nonetheless, they underscore that conventional hormonal regimens may not adequately address the burden of daily pain in endometriosis, further supporting the potential value of adjunctive approaches such as cannabinoid therapy.

### Improvements in fatigue, anxiety, central sensitization, and disability

Endometriosis is increasingly recognized as a systemic condition, with overlapping features of fatigue, mood disturbances, central sensitization, and disability. Treatment with oral CBMPs was associated with significant improvements across these domains.

**Fig 2:**
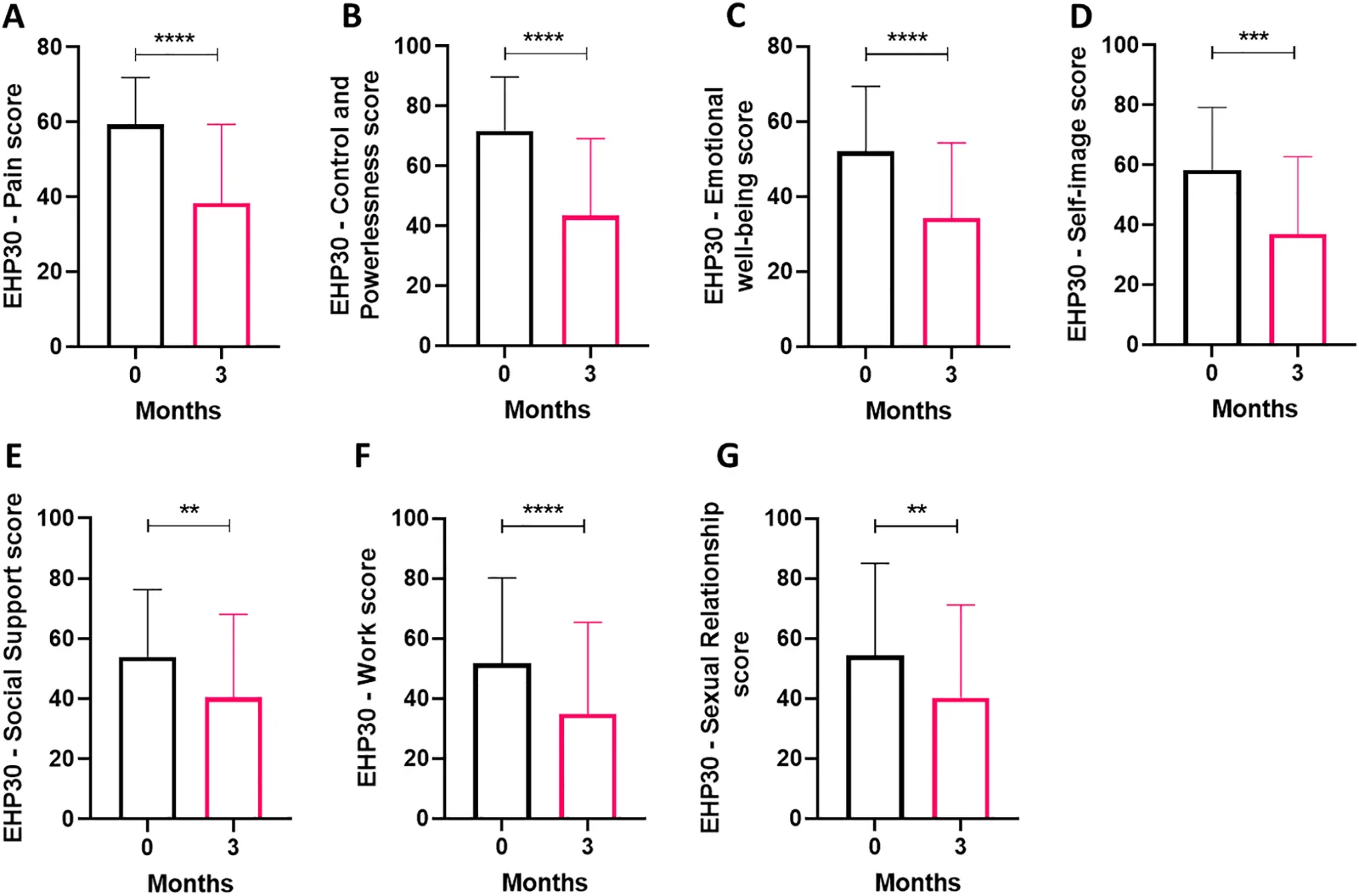
The use of oral CBMPs is associated with improvements in fatigue, anxiety, central sensitization, and pain-related disability in women with endometriosis (Paired t-test). **(A) Fatigue symptoms** significantly decreased after 3 months of treatment, as evidenced by reductions in the Chalder Fatigue Scale (CFS) score (p = 0.0013). **(B) Anxiety severity,** measured by the Generalized Anxiety Disorder 7-item scale (GAD-7), also improved significantly over the treatment period (p < 0.0001). **(C) Central sensitization,** assessed via the Central Sensitization Inventory (CSI), was markedly reduced following treatment (p < 0.0001). **(D) Pain disability** decreased significantly, as shown by lower scores on the Pain Disability Index (PDI; p < 0.0001).

Fatigue symptoms improved after 3 months, as demonstrated by reductions in the CFS score (Fig 2A; p = 0.0013). This effect may relate to cannabinoids’ modulatory influence on sleep regulation, inflammation, and energy balance, providing relief in an often-overlooked dimension of endometriosis.

Similarly, anxiety severity decreased markedly over the treatment period (GAD-7, Fig 2B; p < 0.0001). Given the high prevalence of anxiety and depression among women with endometriosis, this dual effect on both pain and mood highlights a therapeutic advantage over conventional analgesics, which primarily target nociceptive pathways without addressing psychological comorbidities.

A notable finding was the reduction in central sensitization, measured by the CSI (Fig 2C; p < 0.0001). Since central sensitization is a recognized driver of refractory, treatment-resistant pain, often limiting the effectiveness of NSAIDs and opioids, these results suggest that cannabinoids may act on neurophysiological mechanisms of pain amplification beyond peripheral lesion activity.

Finally, patients reported significant functional improvements. The PDI showed marked reductions (Fig 2D; p < 0.0001), indicating that pain interfered less with daily activities, social engagement, and work performance following treatment. Together, these findings underscore the potential of oral CBMPs to improve not only pain but also the broader symptom burden and quality of life in women with endometriosis.

### Endometriosis health profile-30

The impact of endometriosis on health-related quality of life was assessed using the EHP-30, a validated instrument designed to capture physical, psychological, and social domains specific to the disease. Each domain of the EHP-30 is scored on a 0–100 scale, where 0 represents the best health status and 100 the worst possible status, meaning higher values indicate greater disease burden. At baseline, participants reported a considerable burden of symptoms, reflecting severe pain, impaired physical functioning, emotional distress, and restrictions in social and professional life. After three months of treatment with oral CBMPs, EHP-30 outcomes demonstrated a clear and statistically significant reduction in the disease-specific burden, with improvements observed across all domains.

**Fig 3:**
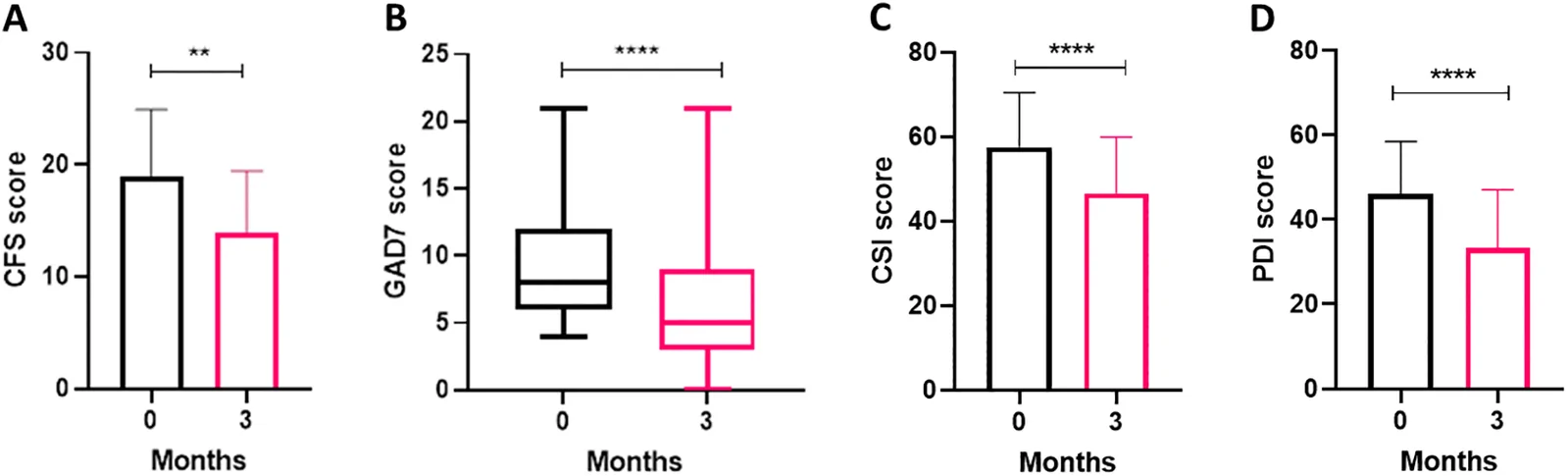
Impact of oral CBMPs on endometriosis-related quality of life assessed by EHP-30. The Endometriosis Health Profile-30 (EHP-30) was used to evaluate disease-specific health-related quality of life across multiple domains, with scores ranging from 0 (best health status) to 100 (worst health status). Participants exhibited substantial baseline disease burden, which was significantly reduced after three months of treatment. **(A) Pain scores** decreased from 59.34 ± 12.58 to 38.38 ± 21.05 (p < 0.0001). **(B) Sense of control and powerlessness** improved from 72.76 ± 17.92 to 43.52 ± 25.72 (p < 0.0001)**. (C) Emotional well-being scores** decreased from 52.01 ± 17.39 to 34.26 ± 20.03 (p < 0.0001). (D) Self-image improved from 58.33 ± 20.93 to 37.04 ± 25.77 (p < 0.001), suggesting enhanced body image and reduced feelings of inadequacy. **(E) Social support** decreased from 53.94 ± 22.34 to 40.51 ± 27.54 (p < 0.01). **(F) Work-related impairment** decreased from 51.67 ± 28.52 to 34.81 ± 30.56 (p < 0.0001). **(G) Sexual relationships** improved from 54.26 ± 30.81 to 40.19 ± 30.99 (p < 0.01). Data are presented as mean ± SD. Statistical comparisons were performed using paired t-tests.

Scores in the pain domain decreased from 59.34 ± 12.58 to 38.38 ± 21.05 (Fig 3A; p < 0.0001), indicating better symptom control and physical functioning. Similarly, the sense of control and powerlessness improved markedly, with scores falling from 72.76 ± 17.92 to 43.52 ± 25.72 (Fig 3B; p < 0.0001), suggesting that patients felt more autonomous and resilient in coping with their condition. Emotional well-being also improved significantly, with scores decreasing an average of 17.75 points (Fig 3C; from 52.01 ± 17.39 to 34.26 ± 20.03; p < 0.0001), reflecting reductions in distress, frustration, and mood-related burden. Self-image scores improved from 58.33 ± 20.93 to 37.04 ± 25.77 (Fig 3D; p < 0.001), indicating enhanced body image and fewer feelings of inadequacy, while social support improved from 53.94 ± 22.34 to 40.51 ± 27.54 (Fig 3E; p < 0.01), suggesting a stronger perception of support from family, friends, or peers. Work-related impairment was also reduced by 16.85 points in the score (Fig 3F; from 51.67 ± 28.52 to 34.81 ± 30.56; p < 0.0001), highlighting diminished interference of endometriosis with professional activities. Finally, sexual relationships improved from 54.26 ± 30.81 to 40.19 ± 30.99 (Fig 3G; p < 0.01), demonstrating that cannabinoid treatment alleviated some of the negative consequences of endometriosis on intimacy and sexual health.

## Discussion

This prospective, observational study demonstrates that oral CBMPs are associated with clinically meaningful improvements in pain intensity, frequency, and analgesic use in women with endometriosis. Over three months, participants experienced not only reductions in pelvic pain but also improvements in fatigue, anxiety, central sensitization, and functional/pain disability, highlighting the potential of cannabinoids to address both nociceptive and neuropsychological dimensions of endometriosis.

Our findings align with preclinical evidence showing that cannabinoids modulate endometrial lesion proliferation, inflammation, and neurogenic sensitization via the ECS [19–21,23]. Through its agonistic effect on the cannabinoid receptor 1 (CB1) and 2 (CB2), THC reduces neurotransmitter release (particularly glutamatergic activity) in the nociceptive system, thereby exerting analgesic effects [25]. In addition to its activity at CB1 and CB2 receptors, THC also interacts with transient receptor potential (TRP) channels such as TRPV2-4, TRPM8, and TRPA1, and slightly inhibits COX-2. These combined mechanisms may contribute to its analgesic, anti-inflammatory, and antineuropathic properties [25–27]. CBD acts largely independently of CB1 and CB2, but can activate TRPV1-3 and TRPA1 channels, and enhance (at high doses) the endocannabinoid tone of anandamide (AEA) by inhibiting its degradation enzyme FAAH. Both mechanisms may contribute to its analgesic effects [25,28]. Beyond CB1, CB2, and TRP channels (TRPV, TRPA, and TRPM subfamilies), cannabinoids also interact with a range of other receptors, including GPR55, GPR18, intracellular PPARs, α3 glycine receptors, and several G-protein-coupled receptors such as opioid and serotonin (5-HT) receptors [28–30]. By engaging these diverse receptor systems expressed along peripheral and central pain pathways, THC and CBD have the potential to attenuate nociceptive signaling and reduce hyperalgesia [16–20]. The observed reduction in central sensitization, as reflected by the CSI scores, is particularly noteworthy, as sensitization contributes to persistent, treatment-resistant pain in endometriosis and is often inadequately addressed by conventional analgesics [1]. This finding holds significant clinical relevance, as the CSI captures a broader neurophysiological construct than momentary pain intensity alone. The VAS reflects only immediate subjective pain severity, whereas the CSI encompasses widespread hypersensitivity, allodynia, cognitive-affective symptoms, and fatigue. Notably, although improvements were seen in both CSI and VAS scores over the treatment period, their parallel reductions do not indicate that they are interchangeable. To further clarify this relationship, a correlation analysis revealed a moderate positive association (Pearson’s r = 0.43; Spearman’s ρ = 0.41, p = 0.0024), confirming a statistically significant but only partial connection between the measures. Therefore, improvements in central sensitization were not simply a proxy for reduced acute pain intensity.The rapid onset of pain relief, evident within the first month, suggests that cannabinoids may exert both early analgesic effects and longer-term modulatory actions on inflammatory and neurophysiological mechanisms. Additionally, the significant decrease in analgesic use indicates a potential opioid- and NSAID-sparing effect, which could reduce medication-related adverse events and healthcare costs. This is consistent with a growing body of evidence supporting cannabinoids’ medical potential as a complementary or alternative treatment for opioid addiction [31].

Improvements in fatigue and anxiety further underscore the holistic impact of cannabinoid therapy [32]. These effects may be mediated through ECS interactions with neurotransmitter systems involved in mood, sleep, and stress responses [15,16]. The dual action on pain and pain-related psychological symptoms may offer an advantage over conventional pharmacotherapy, which often fails to address comorbid anxiety or chronic fatigue.

Despite these promising results, several limitations must be acknowledged. The study’s small sample size and single-center design limit generalizability, and the observational nature precludes definitive causal inferences. Reliance on self-reported measures introduces the possibility of expectation or reporting bias, and the lack of a placebo control prevents differentiation of treatment-specific effects from nonspecific improvements.

The placebo effect is particularly relevant in chronic pain conditions such as endometriosis, where responses can be substantial, especially among patients who have exhausted conventional treatment options. The open-label design, self-selected population, and reliance on patient-reported outcomes further increase susceptibility to expectation bias. Selection bias is a further concern: patients who seek out cannabinoid treatment after failing conventional therapies may carry heightened expectations toward this intervention, contributing to expectation-driven improvements beyond any pharmacological effect. Participants were not randomly recruited from the general endometriosis population but were offered inclusion based on eligibility criteria, introducing a degree of self-selection that limits the representativeness of the sample. Nevertheless, the observation of concurrent improvements across multiple validated instruments capturing distinct domains, the stability of doses without escalation across three months, and the reduction in central sensitization scores may suggest a component beyond purely expectation-driven effects. However, this interpretation remains indirect, and randomized placebo-controlled trials are required to determine the true magnitude of treatment-specific effects.

Additionally, the relatively short follow-up period does not allow assessment of long-term efficacy, tolerance, or safety. Within this study, no evidence of pharmacological tolerance emerged — doses remained stable and clinical gains were sustained or continued to grow across all three months. However, chronic THC exposure is known to produce CB1 receptor downregulation and desensitization, with potential tolerance to some effects and withdrawal challenges upon discontinuation. Longer-term effects on cognition, hormonal regulation, and endocannabinoid system homeostasis remain incompletely characterized, particularly in reproductive-age women. Future studies must therefore include longer follow-up windows and systematic safety monitoring protocols targeting these domains.

Future randomized controlled trials with larger cohorts and longer follow-up are required to confirm these preliminary findings.

In conclusion, oral CBMPs with balanced THC: CBD ratios seem to be a safe and potentially effective adjunct therapy for chronic pelvic pain and related symptoms in women with endometriosis. By targeting both nociceptive and neuropsychological aspects of the condition, CBMPs can supplement existing pharmacological and lifestyle strategies, providing a more comprehensive approach to symptom management. These findings establish a basis for future controlled studies to refine dosing, investigate mechanisms, and assess long-term outcomes in this complex patient group.

## Supporting information

S1 Table Frequency and severity of reported side effects. (DOCX)

S2 File Complete dataset. Complete dataset from the study.
(ZIP)

S3 File STROBE. STROBE-checklist cohort.
(PDF)
