# Supplementary material for: Evaluating the impact of cannabinoid extracts on chronic pain and quality of life in endometriosis patients – a pilot study
Source: PLoS One. 2026 Sep 22;21(9):e0356768. doi: 10.1371/journal.pone.0356768 (PMC13596799; doi:10.1371/journal.pone.0356768)
Supplement: Supplementary file 1 [file pone.0356768.s003.pdf]

STROBE Statement—Checklist of items that should be included in reports of ***cohort studies***

	Item No	Recommendation
**Title and abstract**	1	(*a*) Indicate the study's design with a commonly used term in the title or the abstract<br>Yes. The abstract explicitly states: "This prospective single-center cohort study…"
		(*b*) Provide in the abstract an informative and balanced summary of what was done and what was found<br>Yes. The abstract summarizes background, methods, population, intervention, outcomes, results, limitations, and conclusions.
**Introduction**		
Background/rationale	2	Explain the scientific background and rationale for the investigation being reported<br>Yes. Scientific background and rationale are clearly described, including unmet needs in endometriosis pain management and the biological plausibility of cannabinoids.
Objectives	3	State specific objectives, including any prespecified hypotheses<br>Yes. The objective is explicitly stated: evaluation of the effects of oral CBMPs on pain, symptom burden, and quality of life.<br>∗ Prespecified primary and secondary outcomes are defined in Methods.
**Methods**		
Study design	4	Present key elements of study design early in the paper<br>Yes. Described early as a prospective, single-center cohort study.
Setting	5	Describe the setting, locations, and relevant dates, including periods of recruitment, exposure, follow-up, and data collection<br>Yes.<br>Location: Endometriosis Center Charité, Berlin, Germany<br>Recruitment: November 2023 – May 2025<br>Follow-up: 3 months<br>Data collection: online questionnaires at baseline and months 1–3
Participants	6	(*a*) Give the eligibility criteria, and the sources and methods of selection of participants. Describe methods of follow-up<br>Yes.<br>Inclusion and exclusion criteria are fully described<br>Source: clinical population at Charité<br>Follow-up method: online questionnaires
		(*b*) For matched studies, give matching criteria and number of exposed and unexposed<br>Not applicable (no matched groups)
Variables	7	Clearly define all outcomes, exposures, predictors, potential confounders, and effect modifiers. Give diagnostic criteria, if applicable<br>Yes.<br>Outcomes: pain (VAS), EHP-30, PDI, CFS, GAD-7, CSI<br>Exposure: oral CBMPs (THC: CBD balanced extract)<br>Confounders described descriptively (e.g., hormone therapy, prior treatments)
Data sources/ measurement	8*	For each variable of interest, give sources of data and details of methods of assessment (measurement). Describe comparability of assessment methods if there is more than one group<br>Yes.<br>Validated questionnaires specified<br>Same assessment methods used for all participants

Bias	9	Describe any efforts to address potential sources of bias<br>Expectation bias, self-reporting bias, lack of placebo control, and observational design are discussed in the Discussion section.<br>No formal bias mitigation strategy beyond standardized instruments.
Study size	10	Explain how the study size was arrived at<br>Yes.<br>Defined as a pilot study<br>Sample size based on feasibility, not formal power calculation
Quantitative variables	11	Explain how quantitative variables were handled in the analyses. If applicable, describe which groupings were chosen and why<br>Yes.<br>Continuous variables analyzed as means ± SD or medians (IQR)<br>Groupings (dose tertiles) explained
Statistical methods	12	(*a*) Describe all statistical methods, including those used to control for confounding<br>Yes.<br>Paired tests, Friedman test with post hoc correction<br>No adjustment for confounders (appropriate for pilot cohort)
		(*b*) Describe any methods used to examine subgroups and interactions<br>Yes.<br>Exploratory analyses by dose tertiles and hormone use
		(*c*) Explain how missing data were addressed<br>Yes.<br>Loss to follow-up reported<br>Participants with missing questionnaires excluded from final analysis
		(*d*) If applicable, explain how loss to follow-up was addressed<br>Yes.<br>3/30 participants lost; reasons stated
		(*e*) Describe any sensitivity analyses<br>Not performed (acceptable for pilot study)
**Results**		
Participants	13*	(a) Report numbers of individuals at each stage of study—eg numbers potentially eligible, examined for eligibility, confirmed eligible, included in the study, completing follow-up, and analysed<br>Yes.<br>Enrolled: 30<br>Completed: 27<br>Reasons for exclusion provided
		(b) Give reasons for non-participation at each stage<br>Yes.<br>Side effects (n=1)<br>Missing questionnaires (n=2)
		(c) Consider use of a flow diagram<br>Not included.
Descriptive data	14*	(a) Give characteristics of study participants (eg demographic, clinical, social) and information on exposures and potential confounders<br>Yes. Demographics, disease stage, treatments, prior surgeries, cannabis exposure
		(b) Indicate number of participants with missing data for each variable of interest<br>Not explicitly tabulated.
		(c) Summarise follow-up time (eg, average and total amount)

		Yes. 3 months for all analyzed participants
Outcome data	15*	Report numbers of outcome events or summary measures over time<br>Yes. Pain, symptom scores, and QoL outcomes reported over time
Main results	16	(*a*) Give unadjusted estimates and, if applicable, confounder-adjusted estimates and their precision (eg, 95% confidence interval). Make clear which confounders were adjusted for and why they were included<br>Yes. Unadjusted estimates with p-values reported; No adjusted estimates (appropriate)
		(*b*) Report category boundaries when continuous variables were categorized<br>Yes (dose tertiles defined)
		(*c*) If relevant, consider translating estimates of relative risk into absolute risk for a meaningful time period<br>Not applicable (continuous outcomes)
Other analyses	17	Report other analyses done—eg analyses of subgroups and interactions, and sensitivity analyses<br>Yes. Dose–side effect correlations; Subgroup comparisons
**Discussion**		
Key results	18	Summarise key results with reference to study objectives<br>Yes. Summarized clearly in relation to objectives
Limitations	19	Discuss limitations of the study, taking into account sources of potential bias or imprecision. Discuss both direction and magnitude of any potential bias<br>Yes.<br>Small sample size<br>Observational design<br>No placebo control<br>Short follow-up<br>Self-reporting bias
Interpretation	20	Give a cautious overall interpretation of results considering objectives, limitations, multiplicity of analyses, results from similar studies, and other relevant evidence<br>Yes.<br>Cautious interpretation<br>Positioned as preliminary evidence<br>Compared with existing literature
Generalisability	21	Discuss the generalisability (external validity) of the study results<br>Yes. Limitations to external validity acknowledged
**Other information**		
Funding	22	Give the source of funding and the role of the funders for the present study and, if applicable, for the original study on which the present article is based<br>Yes. No funding received; Product provision disclosed; Role of funders clarified

*Give information separately for exposed and unexposed groups.
