# Supplementary material for: Evaluating the impact of cannabinoid extracts on chronic pain and quality of life in endometriosis patients – a pilot study
Source: PLoS One. 2026 Sep 22;21(9):e0356768. doi: 10.1371/journal.pone.0356768 (PMC13596799; doi:10.1371/journal.pone.0356768)
Supplement: Supplementary file 2 [file pone.0356768.s001.docx]

**Supplemental Material 1. Frequency and Severity of Reported Side Effects**

| ***Side effect*** | ***1-month follow-up*** | | ***2 months follow-up*** | | ***3 months follow-up*** | |
| --- | --- | --- | --- | --- | --- | --- |
|  | ***Prevalence (%)*** | ***Median (IQR)*** | ***Prevalence (%)*** | ***Median (IQR)*** | ***Prevalence (%)*** | ***Median (IQR)*** |
| Fatigue/ Tiredness | 74.1 | 2 (0.5–2) | 74.1 | 1 (0.5–2) | 66.7 | 1 (0–2) |
| Dry mouth | 59.3 | 1 (0–1.5) | 63 | 1 (0–1) | 40.7 | 0 (0–1) |
| Red eyes | 25.9 | 0 (0–0.5) | 29.6 | 0 (0–1) | 25.9 | 0 (0–0.5) |
| Dizziness | 48.1 | 0 (0–1) | 48.1 | 0 (0–1) | 33.3 | 0 (0–1) |
| Nausea | 29.6 | 0 (0–1) | 25.9 | 0 (0–0.5) | 14.8 | 0 (0–0) |
| Motor function / Coordination | 40.7 | 0 (0–1) | 55.6 | 1 (0–1) | 29.6 | 0 (0–1) |
| Feeling high / Intoxicated | 29.6 | 0 (0–1) | 48.1 | 0 (0–1) | 37 | 0 (0–1) |
| Concentration (or difficulty concentrating) | 59.3 | 1 (0–1) | 70.4 | 1 (0–2) | 63 | 1 (0–2) |
| Memory (or memory impairment) | 55.6 | 1 (0–1) | 59.3 | 1 (0–2) | 74.1 | 1 (0.5–1) |
| Panic/ Anxiety | 25.9 | 0 (0–0.5) | 22.2 | 0 (0–0) | 3.7 | 0 (0–0) |
| Paranoia | 3.7 | 0 (0–0) | 0 | 0 (0–0) | 0 | 0 (0–0) |
| Heart rate | 18.5 | 0 (0–0) | 18.5 | 0 (0–0) | 11.1 | 0 (0–0) |
| Appetite | 44.4 | 0 (0–1) | 55.6 | 1 (0–1) | 40.7 | 0 (0–1) |
| Other | 14.8 | 0 (0–0) | 25.9 | 0 (0–0.5) | 11.1 | 0 (0–0) |
| Restlessness/ Agitation | 18.5 | 0 (0–0) | 48.1 | 0 (0–1) | 33.3 | 0 (0–1) |
