# Supplementary material for: Evaluating the impact of cannabinoid extracts on chronic pain and quality of life in endometriosis patients – a pilot study
Source: PLoS One. 2026 Sep 22;21(9):e0356768. doi: 10.1371/journal.pone.0356768 (PMC13596799; doi:10.1371/journal.pone.0356768)
Supplement: Supplementary file 3 [file pone.0356768.s002.zip › Baseline (ENDOCAN) en-US.pdf]

Subscribe to DeepL Pro to translate larger documents.
Visit www.DeepL.com/pro for more information.

ENDOCAN → base September 3, 2024,
12:53 p.m.

BL01

BASELINE QUESTIONNAIRE for the ENDOCAN Study

Dear Patient,

Your doctor has asked you if you would like to participate in an observational study on the improvement of endometriosis-related symptoms through the use of cannabis extracts. To gain important new insights into the treatment of endometriosis and thereby improve the situation for those affected, we ask that you answer the following questionnaires carefully, on time (before the start of treatment, and after 1, 2, and 3 months of treatment), and truthfully. Please take your time when answering the questions. We are available to answer any questions you may have at any time (janosch-willem.kratz@charite.de).

BL02

1. Please provide us with the following information:

First Name:

Last name:

Date of birth:

Email

BL03

2. Have you already been diagnosed with endometriosis?

- ◯ No
- ◯ Yes, in what year?

BL04

3. Age at first menstrual period:

- ◯ years
- ◯ Unknown

BL05

4. First day of the last menstrual period:

- ◯ Date:
- ◯ Currently not menstruating
- ◯ Unknown

1 active filter

Filter BL05/F1
If one of the following answer options was selected: **2** Then hide page(s) **jump1** of the questionnaire

BL06

5. Is menstrual bleeding regular (every 25–35 days):

- ◯ yes
- ◯ no
- ◯ unknown
- ◯ all ____ days

BL07

6. How heavy is your average period:

- ◯ heavy
- ◯ moderate
- ◯ light
- ◯ no bleeding

BL08

7. On average, how many days does your menstrual bleeding last?

- ◯ ____ Days
- ◯ Unknown
- ◯ No bleeding at the moment

Page 03

BL09

8. Are you currently taking the pill or using any other hormonal contraceptives? (Pill/hormonal IUD/patch/ring)

- ◯ Yes, I am taking the following medication ____
- ◯ . No
- ◯ Unknown

1 active filter

Filter BL09/F1
If one of the following answer options was selected: **1**
Then display question/text **BL10** later in the questionnaire (otherwise hide it)

BL10

9. How do you take the hormone medication?

◯ Cyclically, i.e., for 21 (24) days, followed by a break or inactive pills◯
Break every ______ months
◯ continuously, without a break

BL11

10. Which of the following medications have you taken so far to treat your endometriosis-related pain?

Diclofenac (e.g., Voltaren© tablets)	I have never taken it	I have taken it in the past	I am currently taking it
Buscopan Plus (tablet, coated tablet)	I have never taken	I have taken it in the past	I am currently taking it
Buscopan Plus (suppositories)	I have never taken	I have taken it in the past	I am currently taking it
Acetylsalicylic acid/ASA (e.g., Aspirin©)	I have never taken	I have taken it in the past	I am currently taking it
Celecoxib (e.g., Celebrex©)	I have never taken	I have taken it in the past	I am currently taking it
Ibuprofen	I have never taken	I have taken it in the past	I am currently taking it
Acetaminophen	I have never taken	I have taken it in the past	I am currently taking it
Metamizole (Novaminsulfon©)	I have never taken it	I have taken it in the past	I am currently taking it
Tramadol (e.g., Tramal©)	I have never taken	I have taken it in the past	I am currently taking it
Tilidine	I have never taken	I have taken it in the past	I am currently taking it
Dihydrocodeine	I have never taken	I have taken it in the past	I am currently taking it
Morphine	I have never taken	I have taken it in the past	I am currently taking it
Oxycodone	I have never taken	I have taken it in the past	I am currently taking it
Levomethadone	I have never taken	I have taken it in the past	I am currently taking it
Fentanyl	I have never taken	I have taken it in the past	I am currently taking it
Pethidine	I have never taken	I have taken it in the past	I am currently taking it
Buprenorphine	I have never taken	I have taken it in the past	I am currently taking it

Piritramide	I have never taken	I have taken it in the past	I am currently taking it
Co-analgesics such as amitriptyline, doxepin, clomipramine, and imipramine	I have never taken these	I have taken them in the past	I am currently taking them
Co-analgesics such as carbamazepine, gabapentin, pregabalin	I have never taken	I have taken them in the past	I am currently taking
Benzodiazepines (diazepam, lorazepam, lormetazepam, midazolam, oxazepam, temazepam, flunitrazepam)	I have never taken	I have taken them in the past	I am currently taking them

11. Have you taken any other pain relievers not listed above?

BL12

12. What complementary therapies have you tried? Did they lead to any improvement?

BL13

	Never Tried	None Improvement	slight Improvement	significant Improvement
Dietary changes Yoga	○	○	○	○
Osteopathy, physical	○	○	○	○
therapy, acupuncture,	○	○	○	○
pelvic floor exercises	○	○	○	○
Psychotherapy / psychological support	○	○	○	○
Sports	○	○	○	○
REHAB	○	○	○	○
	○	○	○	○
	○	○	○	○

Page 05

BL14

13. What is/was the average pain intensity over the past month?

- ◯ 0 (no pain)
- ◯ 1
- ◯ 2
- ◯ 3
- ◯ 4
- ◯ 5
- ◯ 6
- ◯ 7
- ◯ 8
- ◯ 9
- ◯ 10 (the most severe pain imaginable)

BL15

14. On average, how many days a month do you experience pain?

◯ On average, I experience pain [ ] days a month ◯ I have pain every day

◯ I never have pain

BL16

15. On average, how many days a month do you take pain medication?

◯ On average, I take pain relievers [ ] days a month to relieve the pain ◯ I don’t usually take pain relievers

1 active filter

Filter BL16/F1
If any of the following response options were selected: **2**, then hide question/text **BL17** later in the questionnaire

BL17

16. Can the pain be relieved by taking painkillers?

- ◯ Taking pain relievers completely eliminates my pain
- ◯ Despite taking pain relievers, I still experience pain an average of ______ days per month
- ◯ I don’t usually take painkillers

BL18

17. Cannabis use:

	Never used	Tried it once many years ago	I use cannabis a few times a year	I use cannabis occasionally	I use cannabis regularly (several times a week)
Do you use cannabis?					

BL19

18. Have you ever used cannabis to relieve symptoms caused by endometriosis?

- ◯ Yes
- ◯ No
- ◯ Unknown

1 active filter

Filter BL19/F1
If one of the following answer options was selected: **1**
Then display question/text **BL20** later in the questionnaire (otherwise hide it)

Page 07

BL20

19. In what form have you used cannabis to treat your endometriosis-related symptoms?

- ☐ Cannabis flowers without THC (over-the-counter CBD flowers)
- ☐ Cannabis flowers with THC
- ☐ CBD oil
- ☐ Cannabis oil with THC
- ☐ Edibles (cannabis chocolate, cookies, brownies, etc.)

BL21

20. Do you have a family history of endometriosis?

- ◯ Yes, who? [Mother, sister, etc.]
- ◯ No
- ◯ Unknown

BL22

21. Have you ever been pregnant?

- ◯ Yes
- ◯ No
- ◯ Unknown

1 active filter

Filter BL22/F1
If one of the following answer options was selected: **1**
Then display question/text **BL23** later in the questionnaire (otherwise hide it)

BL23

22. If you have ever been pregnant...

☐ Total ☐ Pregnancies

Total Miscarriages

☐ Total Abortions

BL24

23. Have you already had surgery for your endometriosis?

◯ Yes

◯ No

◯ Unknown

1 active filter

Filter BL24/F1
If one of the following answer options was selected: **1** Then display page(s) **jump2** of the questionnaire (otherwise hide)

BL25

24. How many surgeries have you had in total due to your endometriosis?

- ◯ 1 surgery
- ◯ 2 surgeries
- ◯ 3 surgeries
- ◯ 4 Operation
- ◯ > 4 surgeries

BL26

25. Which ASF/rASRM stage were you diagnosed with?

Surgical reports and doctors' letters often specify the ASF/rASRM stage of endometriosis lesions. If you know your ASF/rASRM stage, please indicate it here.

- ☐ No information
- ☐ I
- ☐ II
- ☐ III
- ☐ IV

BL27

26. Which ENZIAN classification was determined for your surgery?

Surgical reports and physician's letters often specify the ENZIAN classification of endometriosis lesions. If you know your classification, please indicate it. (Multiple selections allowed)

☐ A1	☐ A2	☐ A3
☐ B1	☐ B2	☐ B3
☐ C1	☐ C2	☐ C3
☐ P1	☐ P2	☐ P3
☐ O1	☐ O2	☐ O3
☐ T1	☐ T2	☐ T3
☐ FA	☐ FB	☐ FU
☐ FI	☐ FO	☐ Not specified

CF01

27. We’d like to learn more about the problems you’ve had in the past month because you’ve felt tired, weak, or lacking energy. Please answer ALL questions by checking the answer that best applies to you. If you’ve been feeling tired for a long time, compare how you feel now to how you felt when you were last feeling well.

Please check only one box per line.

Do you have problems with fatigue?	less than usual	no more than usual (unchanged)	more than usual	much more than usual
Do you need to rest more often?	less than usual	no more than usual (unchanged)	more than usual	much more than usual
Do you feel tired or sleepy?	less than usual	no more than usual (unchanged)	more than usual	much more than usual
Do you find it hard to get started on tasks?	less than usual	no more than usual (unchanged)	more than usual	much more than usual
Do you feel weak or lacking in energy?	less than usual	no more than usual (unchanged)	more than usual	much more than usual
Do you have less strength in your muscles?	less than usual	no more than usual (unchanged)	more than usual	much more than usual
Do you feel weak?	less than usual	no more than usual (unchanged)	more than usual	much more than usual
Do you have trouble concentrating?	less than usual	no more than usual (unchanged)	more than usual	much more than usual
Do you frequently slip up when speaking?	less than usual	no more than usual (unchanged)	more than usual	much more than usual
Do you find it harder to find the right word?	less than usual	no more than usual (unchanged)	more than usual	much more than usual

CF02

28. Rate your memory performance

How would you rate your memory?	Better than usual	as usual	worse than usual	much worse than usual

29. For each statement, please select the answer on the right that best applies to you at this time.

When I wake up in the morning, I feel tired and not well-rested.	never	Rarely	occasionally	often	always
My muscles feel stiff and sore.	never	Rarely	occasionally	often	always
I have anxiety attacks.	never	Rarely	occasionally	often	always
I grind or clench my teeth.	never	rarely	occasionally	often	always
I have problems with diarrhea and/or constipation.	never	Rarely	occasionally	often	always
I need help with my daily activities.	never	Rarely	occasionally	often	always
I am sensitive to bright light.	never	Rarely	occasionally	often	always
I get tired very quickly during physical activities.	never	Rarely	occasionally	often	always
I have pain all over my body.	never	rarely	occasionally	often	always
I have a headache.	never	rarely	occasionally	often	always
My bladder feels uncomfortable and/or I have a burning sensation when I urinate.	never	Rarely	occasionally	often	always
I don't sleep well.	never	rarely	occasionally	often	always
I have trouble concentrating.	never	rarely	occasionally	often	always
I have skin problems, such as dry or itchy skin or a rash.	never	Rarely	occasionally	often	always
Stress makes my physical symptoms worse.	never	Rarely	occasionally	often	always
I feel sad or down.	never	Rarely	occasionally	often	always
I have little energy.	never	Rarely	occasionally	often	always
I have muscle tension in my neck and shoulders.	never	Rarely	occasionally	often	always
I have a toothache.	never	rarely	occasionally	often	always
Certain smells, such as perfume, make me feel dizzy and nauseous.	never	Rarely	occasionally	often	always
I have to urinate frequently.	never	Rarely	occasionally	often	always
My legs feel uncomfortable and restless when I try to fall asleep at night.	never	Rarely	occasionally	often	always
I have trouble remembering things.	never	rarely	occasionally	often	always
I experienced traumatic events as a child.	never	Rarely	occasionally	often	always
I have pain in my pelvic area.	never	Rarely	occasionally	often	always

EM01

30. How often during the past 4 weeks have you, because of your endometriosis...

been unable to attend social events because of pain?	Never	Rarely	Sometimes	Often	Always
unable to do any housework because of pain?	Never	Rarely	Sometimes	Often	Always
Do you have difficulty standing due to pain?	Never	Rarely	Sometimes	Often	Always
Do you have difficulty sitting because of pain?	Never	Rarely	Sometimes	Often	Always
Do you have difficulty walking due to pain?	Never	Rarely	Sometimes	Often	Always
Do you find it difficult to exercise or engage in the leisure activities you'd like to do because of pain?	Never	Rarely	Sometimes	Often	Always
lost your appetite and/or been unable to eat because of pain?	Never	Rarely	Sometimes	Often	Always
unable to sleep well because of pain?	Never	Rarely	Sometimes	Often	Always
Do you have to go to bed or lie down because of pain?	Never	Rarely	Sometimes	Often	Always
unable to do the things you wanted to do because of pain?	Never	Rarely	Sometimes	Often	Always
felt unable to cope with the pain?	Never	Rarely	Sometimes	Often	Always
Do you generally feel unwell?	Never	Rarely	Sometimes	Often	Always
felt frustrated because your symptoms aren't getting better?	Never	Rarely	Sometimes	Often	Always
felt frustrated because you can't control your symptoms?	Never	Rarely	Sometimes	Often	Always
Have you ever felt unable to forget the symptoms?	Never	Rarely	Sometimes	Often	Always
felt that your symptoms are controlling your life?	Never	Rarely	Sometimes	Often	Always
felt like your symptoms were taking your life away?	Never	Rarely	Sometimes	Often	Always
felt down?	Never	Rarely	Sometimes	Often	Always
felt tearful or close to tears?	Never	Rarely	Sometimes	Frequently	Always
felt miserable?	Never	Rarely	Sometimes	Often	Always
Have you ever suffered from mood swings?	Never	Rarely	Sometimes	Often	Always
felt in a bad mood or irritable?	Never	Rarely	Sometimes	Often	Always
felt hostile or aggressive?	Never	Rarely	Sometimes	Often	Always
Have you ever felt unable to explain to others how you feel?	Never	Rarely	Sometimes	Often	Always
felt that others don't understand what you're going through?	Never	Rarely	Sometimes	Often	Always

felt that others think you're complaining?	Never	Rarely	Sometimes	Often	Always
Alone?	Never	Rarely	Sometimes	Often	Always
felt frustrated because you can't always wear the clothes you'd like to choose?	Never	Rarely	Sometimes	Often	Always
felt that your appearance was affected?	Never	Rarely	Sometimes	Often	Always
had less self-confidence?	Never	Rarely	Sometimes	Often	Always

EM02

31. How often during the past 4 weeks have you, because of your endometriosis...

had to take time off work?	Never	Rarely	Sometimes	Often	Always
unable to perform your job duties?	Never	Rarely	Sometimes	Often	Always
felt ashamed or guilty at work because of your symptoms?	Never	Rarely	Sometimes	Often	Always
Do you feel guilty because you took time off from work?	Never	Rarely	Sometimes	Often	Always
Have you ever been afraid that you wouldn't be able to do your job?	Never	Rarely	Sometimes	Often	Always

EM03

32. How often during the past 4 weeks have you, because of your endometriosis...

experienced pain during or after sexual intercourse?	Never	Rarely	Sometimes	Often	Always
Have you ever been worried about having sexual intercourse?	Never	Rarely	Sometimes	Often	Always
Have you avoided sexual intercourse because of the pain?	Never	Rarely	Sometimes	Often	Always
felt guilty because you didn't want to have sexual intercourse?	Never	Rarely	Sometimes	Often	Always
felt frustrated because you can't enjoy sexual intercourse?	Never	Rarely	Sometimes	Often	Always

Page 13

GA01

33. How often have you felt impaired by the following symptoms over the past 4 weeks?

	Not at all	On some days	On more than half the days	Almost every day
Nervousness, anxiety, or tension	○	○	○	○
Being unable to stop or control your worries Excessive worry	○	○	○	○
about various matters Difficulty relaxing	○	○	○	○
Restlessness, making it hard to sit still	○	○	○	○
Getting annoyed or irritated easily	○	○	○	○
A feeling of fear, as if something bad were going to happen	○	○	○	○
	○	○	○	○

34. Please indicate below to what extent your pain in the various areas PD02
of your life. In other words: To what extent does the pain prevent you from leading a normal life? For each of the seven areas of life, please check the number that best describes the typical severity of the impairment caused by your pain. A score of 0 means no impairment at all, and a score of 10 indicates that you are completely impaired in this area due to the pain.

Family and household responsibilities

(This category refers to activities related to the home or the family. It includes housework and activities around the house or apartment, as well as yard work).

	0	1	2	3	4	5	6	7	8	9	10	
no impairment	○	○	○	○	○	○	○	○	○	○	○	Complete impairment

Recreation

(This category includes hobbies, sports, and leisure activities)

	0	1	2	3	4	5	6	7	8	9	10	
none Impairment	○	○	○	○	○	○	○	○	○	○	○	All Impairment

Social Activities

(This section refers to spending time with friends and acquaintances, such as parties, going to the theater or concerts, dining out, and other social activities)

	0	1	2	3	4	5	6	7	8	9	10	
no impairment	○	○	○	○	○	○	○	○	○	○	○	Complete impairment

Work

(This area refers to activities that are part of the job or are directly related to the occupation; this also includes household activities)

	0	1	2	3	4	5	6	7	8	9	10	
none impairment	○	○	○	○	○	○	○	○	○	○	○	Complete impairment

Sexual life

(this area refers to Frequency and quality of sexual life)

	0	1	2	3	4	5	6	7	8	9	10	
none Impairment	○	○	○	○	○	○	○	○	○	○	○	directly Impairment

Self-care

(This area encompasses activities that enable autonomy and independence in daily life, such as washing and dressing oneself, or driving a car, without having to rely on outside help)

	0	1	2	3	4	5	6	7	8	9	10	
no impairment	○	○	○	○	○	○	○	○	○	○	○	complete impairment

Activities Essential for Daily Living

(This category refers to absolutely

(activities essential for life, such as eating, sleeping, and breathing)

	0	1	2	3	4	5	6	7	8	9	10	
none Impairment	○	○	○	○	○	○	○	○	○	○	○	Compl ete Impairment

Thank you very much for your participation!

We would like to express our sincere gratitude for your assistance.

If you have any questions, please feel free to contact us at any time (janosch-willem.kratz@charite.de). Your answers have been saved; you may now close the browser window.

Janosch Kratz – 2023
