# Supplementary material for: Evaluating the impact of cannabinoid extracts on chronic pain and quality of life in endometriosis patients – a pilot study
Source: PLoS One. 2026 Sep 22;21(9):e0356768. doi: 10.1371/journal.pone.0356768 (PMC13596799; doi:10.1371/journal.pone.0356768)
Supplement: Supplementary file 3 [file pone.0356768.s002.zip › Baseline (ENDOCAN).pdf]

soSci
oFb - der onlineFragebogen

ENDOCAN → base 03.09.2024, 12:53

Seite 01
Baseline

BL01

BASELINE-FRAGEBOGEN der ENDOCAN-Studie

Sehr geehrte Patientin,

Sie sind von Ihrem Arzt/Ihrer Ärztin gefragt worden, ob Sie an einer Beobachtungsstudie bezüglich der Verbesserung von endometriosebedingten Symptomen durch die Einnahme von Cannabisextrakten teilnehmen möchten. Um wichtige neue Erkenntnisse bezüglich der Behandlung von Endometriose zu erforschen und somit die Situation für die betroffenen Personen verbessern zu können, bitten wir Sie die folgenden Fragebögen sorgfältig, pünktlich (vor Behandlungsbeginn, nach 1,2 und 3 Monaten Behandlung) und wahrheitsgemäß zu beantworten. Bitte nehmen Sie sich für die Beantwortung ausreichend Zeit. Für Fragen stehen wir Ihnen jederzeit zur Verfügung (janosch-willem.kratz@charite.de).

BL02

1. Bitte teilen Sie uns die folgenden Informationen mit:

Vorname:

Nachname:

Geburtsdatum:

Email

BL03

2. Ist bei Ihnen Endometriose bereits diagnostiziert worden?

- ◯ nein
- ◯ ja, in welchem Jahr?

BL04

3. Alter bei erster Menstruationsblutung:

- ◯ Jahre
- ◯ unbekannt

BL05

4. Erster Tag der letzten Menstruationsblutung:

- ◯ Datum:
- ◯ momentan blutungsfrei
- ◯ unbekannt

1 aktive(r) Filter

Filter BL05/F1
Wenn eine der folgenden Antwortoption(en) ausgewählt wurde: **2**
Dann Seite(n) **jump1** des Fragebogens ausblenden

Seite 02

jump1

BL06

5. Ist die Menstruationsblutung regelmäßig (alle 25-35 Tage):

- ◯ ja
- ◯ nein
- ◯ unbekannt
- ◯ alle [ ] Tage

BL07

6. Wie ist die Stärke ihrer durchschnittlichen Regelblutung:

- ◯ stark
- ◯ mittel
- ◯ schwach
- ◯ keine Blutung

BL08

7. Wieviele Tage hält die Menstruationsblutung bei Ihnen im Durchschnitt an?

- ◯ [ ] Tage
- ◯ unbekannt
- ◯ momentan keine Blutung

Seite 03

BL09

8. Nehmen Sie aktuell die Pille oder wenden sie sonstige Hormonpräparate an? (Pille/Hormonspirale/ Pflaster/ Ring)

- ◯ ja, ich nehme folgendes Präparat ein [ ]
- ◯ nein
- ◯ unbekannt

1 aktive(r) Filter

Filter BL09/F1
Wenn eine der folgenden Antwortoption(en) ausgewählt wurde: **1**
Dann Frage/Text **BL10** später im Fragebogen anzeigen (sonst ausblenden)

BL10

9. Wie nehmen sie das Hormonpräparat ein?

- ◯ zyklisch, d.h. über 21 (24) Tage, dann Pause oder wirkstofffreie Tabletten
- ◯ Pause alle [ ] Monate
- ◯ kontinuierlich, ohne Pause

BL11

10. Welches der folgenden Medikamente haben Sie bisher eingenommen, um ihre endometriose-bedingten Schmerzen zu behandeln?

Diclofenac (z.B. Voltaren© Tabletten)	Habe ich niemals eingenommen	Habe ich in der Vergangenheit eingenommen	Nehme ich aktuell ein
Buscopan Plus (Tablette, Dragee)	Habe ich niemals eingenommen	Habe ich in der Vergangenheit eingenommen	Nehme ich aktuell ein
Buscopan Plus (Zäpfchen)	Habe ich niemals eingenommen	Habe ich in der Vergangenheit eingenommen	Nehme ich aktuell ein
Acetylsalicylsäure/ASS (z.B. Aspirin©)	Habe ich niemals eingenommen	Habe ich in der Vergangenheit eingenommen	Nehme ich aktuell ein
Celecoxib (z.B. Celebrex©)	Habe ich niemals eingenommen	Habe ich in der Vergangenheit eingenommen	Nehme ich aktuell ein
Ibuprofen	Habe ich niemals eingenommen	Habe ich in der Vergangenheit eingenommen	Nehme ich aktuell ein
Paracetamol	Habe ich niemals eingenommen	Habe ich in der Vergangenheit eingenommen	Nehme ich aktuell ein
Metamizol (Novaminsulfon©)	Habe ich niemals eingenommen	Habe ich in der Vergangenheit eingenommen	Nehme ich aktuell ein
Tramadol (z.B. Tramal©)	Habe ich niemals eingenommen	Habe ich in der Vergangenheit eingenommen	Nehme ich aktuell ein
Tilidin	Habe ich niemals eingenommen	Habe ich in der Vergangenheit eingenommen	Nehme ich aktuell ein
Dihydrocodein	Habe ich niemals eingenommen	Habe ich in der Vergangenheit eingenommen	Nehme ich aktuell ein
Morphin	Habe ich niemals eingenommen	Habe ich in der Vergangenheit eingenommen	Nehme ich aktuell ein
Oxycodon	Habe ich niemals eingenommen	Habe ich in der Vergangenheit eingenommen	Nehme ich aktuell ein
Levomethadon	Habe ich niemals eingenommen	Habe ich in der Vergangenheit eingenommen	Nehme ich aktuell ein
Fentanyl	Habe ich niemals eingenommen	Habe ich in der Vergangenheit eingenommen	Nehme ich aktuell ein
Pethidin	Habe ich niemals eingenommen	Habe ich in der Vergangenheit eingenommen	Nehme ich aktuell ein
Buprenorphin	Habe ich niemals eingenommen	Habe ich in der Vergangenheit eingenommen	Nehme ich aktuell ein

Piritramid	Habe ich niemals eingenommen	Habe ich in der Vergangenheit eingenommen	Nehme ich aktuell ein
Koanalgetika wie Amitriptylin, Doxepin, Clomipramin, Imipramin	Habe ich niemals eingenommen	Habe ich in der Vergangenheit eingenommen	Nehme ich aktuell ein
Ko-Analgetika wie Carbamazepin, Gabapentin, Pregabalin	Habe ich niemals eingenommen	Habe ich in der Vergangenheit eingenommen	Nehme ich aktuell ein
Benzodiazepine (Diazepam, Lorazepam, Lormetazepam, Midazolam, Oxazepam, Temazepam, Flunitrazepam)	Habe ich niemals eingenommen	Habe ich in der Vergangenheit eingenommen	Nehme ich aktuell ein

11. Haben sie weitere Schmerzmittel eingenommen die in der vorherigen Liste nicht aufgeführt sind?

BL12

12. Welche komplementären Verfahren haben Sie schon probiert? Haben diese zu einer Verbesserung geführt?

BL13

	noch nie ausprobiert	keine Besserung	leichte Besserung	deutliche Besserung
Ernährungsumstellung	○	○	○	○
Yoga	○	○	○	○
Osteopathie	○	○	○	○
Physiotherapie	○	○	○	○
Akupunktur	○	○	○	○
Beckenbodenübungen	○	○	○	○
Psychotherapie / psychologische Begleitung	○	○	○	○
Sport	○	○	○	○
REHA	○	○	○	○

Seite 05

13. Wie ist/war die durchschnittliche Schmerzstärke im letzten Monat? BL14

- ◯ 0 (kein Schmerz)
- ◯ 1
- ◯ 2
- ◯ 3
- ◯ 4
- ◯ 5
- ◯ 6
- ◯ 7
- ◯ 8
- ◯ 9
- ◯ 10 (stärkster vorstellbarer Schmerz)

14. An wie vielen Tagen leiden sie durchschnittlich im Monat unter Schmerzen? BL15

- ◯ Ich leide durchschnittlich an ______ Tagen im Monat unter Schmerzen
- ◯ Ich habe jeden Tag Schmerzen
- ◯ Ich habe nie Schmerzen

15. An wie vielen Tagen im Monat nehmen Sie durchschnittlich Schmerzmittel ein? BL16

- ◯ Ich nehme durchschnittlich an ______ Tagen im Monat Schmerzmittel ein um die Schmerzen zu lindern
- ◯ Ich nehme normalerweise keine Schmerzmittel ein

1 aktive(r) Filter

Filter BL16/F1
Wenn eine der folgenden Antwortoption(en) ausgewählt wurde: **2**
Dann Frage/Text **BL17** später im Fragebogen ausblenden

Seite 06

16. Können die Schmerzen durch die Einnahme von Schmerzmitteln gelindert werden? BL17

- ◯ Durch die Einnahme von Schmerzmitteln habe ich keinerlei Schmerzen mehr
- ◯ Trotz der Einnahme von Schmerzmitteln leide ich immer noch durchschnittlich an ______ Tagen pro Monat unter Schmerzen
- ◯ Ich nehme normalerweise keine Schmerzmittel ein

17. Cannabiskonsum: BL18

Konsumieren Sie Cannabis?	Noch nie konsumiert	Vor vielen Jahren Mal ausprobiert	Ich konsumiere Cannabis einige wenige Male im Jahr	Ich konsumieren Cannabis gelegentlich	Ich konsumiere Cannabis regelmäßig (merhrmals/Woche)

18. Haben Sie schonmal Cannabis konsumiert, um endometriosebedingte Symptome zu lindern? BL19

- ◯ Ja
- ◯ Nein
- ◯ Unbekannt

1 aktive(r) Filter

Filter BL19/F1
Wenn eine der folgenden Antwortoption(en) ausgewählt wurde: **1**
Dann Frage/Text **BL20** später im Fragebogen anzeigen (sonst ausblenden)

Seite 07

BL20

19. In welcher Form haben Sie Cannabis zur Behandlung ihrer endometriosebedingten Symptome konsumiert?

- ☐ Cannabisblüten ohne THC (frei verkäufliche CBD-Blüten)
- ☐ Cannabisblüten mit THC
- ☐ CBD-Öl
- ☐ Cannabis-Öl mit THC
- ☐ Edibles (Cannabisschokolade, Cookies, Brownies, etc.)

BL21

20. Ist bei Ihnen einen familiäre Endometriose bekannt?

- ○ ja, wer? Mutter, Schwester, etc.
- ○ nein
- ○ unbekannt

BL22

21. Waren Sie jemals schwanger?

- ○ ja
- ○ nein
- ○ unbekannt

1 aktive(r) Filter

Filter BL22/F1
Wenn eine der folgenden Antwortoption(en) ausgewählt wurde: **1**
Dann Frage/Text **BL23** später im Fragebogen anzeigen (sonst ausblenden)

Seite 08
jump2

22. Falls sie jemals schwanger waren...

BL23

- ☐ Insgesamt ___ Schwangerschaften
- ☐ Insgesamt ___ Fehlgeburten
- ☐ Insgesamt ___ Abtreibungen

23. Wurden Sie bereits aufgrund ihrer Endometriose operiert?

BL24

- ◯ ja
- ◯ nein
- ◯ unbekannt

1 aktive(r) Filter

Filter BL24/F1
Wenn eine der folgenden Antwortoption(en) ausgewählt wurde: **1**
Dann Seite(n) **jump2** des Fragebogens anzeigen (sonst ausblenden)

24. Wie viele Operationen hatten Sie bereits insgesamt aufgrund Ihrer Endometriose? BL25

- ◯ 1 Operation
- ◯ 2 Operation
- ◯ 3 Operation
- ◯ 4 Operation
- ◯ > 4 Operationen

25. Welches ASF/rASRM-Stadium wurde bei Ihnen festgestellt? BL26

In OP-Berichten und Arztbriefen wird häufig das ASF/rASRM-Stadium der Endometrioseläsionen angegeben. Falls Ihnen ihr ASF/rASRM-Stadium bekannt ist geben Sie dieses bitte an.

- ☐ Keine angabe
- ☐ I
- ☐ II
- ☐ III
- ☐ IV

26. Welche ENZIAN-Klassifizierung wurde bei Ihrer Operation festgestellt? BL27

In OP-Berichten und Arztbriefen wird häufig die ENZIAN-Klassifizierung von Endometrioseläsionen angegeben. Falls Ihnen ihre Klassifizierung bekannt ist geben Sie diese bitte an. (Mehrfachauswahl möglich)

☐ A1	☐ A2	☐ A3
☐ B1	☐ B2	☐ B3
☐ C1	☐ C2	☐ C3
☐ P1	☐ P2	☐ P3
☐ O1	☐ O2	☐ O3
☐ T1	☐ T2	☐ T3
☐ FA	☐ FB	☐ FU
☐ FI	☐ FO	☐ keine Angabe

CF01

27. Wir würden gerne mehr darüber erfahren, welche Probleme Sie im letzten Monat hatten, weil Sie sich müde, schwach oder energielos fühlten. Bitte beantworten Sie ALLE Fragen, indem Sie die Antwort ankreuzen, die am ehesten auf Sie zutrifft. Wenn Sie sich schon lange müde fühlen, vergleichen Sie sich mit dem Gefühl, als es Ihnen zuletzt gut ging.

Bitte kreuzen Sie nur ein Kästchen pro Zeile an.

Haben Sie Probleme mit Müdigkeit?	weniger als sonst	nicht mehr als üblich (gleichbleibend)	mehr als sonst	viel mehr als sonst
Müssen Sie sich häufiger ausruhen?	weniger als sonst	nicht mehr als üblich (gleichbleibend)	mehr als sonst	viel mehr als sonst
Fühlen Sie sich müde oder schläfrig?	weniger als sonst	nicht mehr als üblich (gleichbleibend)	mehr als sonst	viel mehr als sonst
Fällt es Ihnen schwer, Aufgaben zu beginnen?	weniger als sonst	nicht mehr als üblich (gleichbleibend)	mehr als sonst	viel mehr als sonst
Fühlen Sie sich kraftlos/ohne Energie?	weniger als sonst	nicht mehr als üblich (gleichbleibend)	mehr als sonst	viel mehr als sonst
Haben Sie weniger Kraft in Ihren Muskeln?	weniger als sonst	nicht mehr als üblich (gleichbleibend)	mehr als sonst	viel mehr als sonst
Fühlen Sie sich schwach?	weniger als sonst	nicht mehr als üblich (gleichbleibend)	mehr als sonst	viel mehr als sonst
Haben Sie Konzentrationsschwierigkeiten?	weniger als sonst	nicht mehr als üblich (gleichbleibend)	mehr als sonst	viel mehr als sonst
Machen Sie beim Reden häufig Versprecher?	weniger als sonst	nicht mehr als üblich (gleichbleibend)	mehr als sonst	viel mehr als sonst
Fällt es Ihnen schwerer, das richtige Wort zu finden?	weniger als sonst	nicht mehr als üblich (gleichbleibend)	mehr als sonst	viel mehr als sonst

CF02

28. Beurteilen Sie ihre Gedächtnisleistung

Wie würden Sie ihre Gedächtnisleistung beurteilen?	besser als sonst	wie üblich	schlechter als sonst	viel schlechter als sonst

29. Bitte markieren Sie bei jeder Aussage die für Sie aktuell am besten passende Antwort auf der rechten Seite.

CS01

Wenn ich morgens aufwache, fühle ich mich müde und nicht erholt.	nie	selten	gelegentlich	oft	immer
Meine Muskeln fühlen sich steif und schmerzhaft an.	nie	selten	gelegentlich	oft	immer
Ich habe Angstattacken.	nie	selten	gelegentlich	oft	immer
Ich knirsche oder beiße meine Zähne zusammen.	nie	selten	gelegentlich	oft	immer
Ich habe Probleme mit Durchfall und/oder Verstopfung.	nie	selten	gelegentlich	oft	immer
Ich brauche Hilfe bei der Verrichtung meiner Alltagstätigkeiten.	nie	selten	gelegentlich	oft	immer
Ich reagiere empfindlich auf helles Licht.	nie	selten	gelegentlich	oft	immer
Ich ermüde sehr schnell bei körperlichen Aktivitäten.	nie	selten	gelegentlich	oft	immer
Ich habe am ganzen Körper Schmerzen.	nie	selten	gelegentlich	oft	immer
Ich habe Kopfschmerzen.	nie	selten	gelegentlich	oft	immer
Meine Blase fühlt sich unangenehm an und/oder ich habe Brennen beim Wasserlassen.	nie	selten	gelegentlich	oft	immer
Ich schlafe nicht gut.	nie	selten	gelegentlich	oft	immer
Ich habe Konzentrationsschwierigkeiten.	nie	selten	gelegentlich	oft	immer
Ich habe Hautprobleme, wie z.B. trockene oder juckende Haut oder Hautausschlag.	nie	selten	gelegentlich	oft	immer
Stress verstärkt meine körperlichen Beschwerden.	nie	selten	gelegentlich	oft	immer
Ich fühle mich traurig oder niedergeschlagen.	nie	selten	gelegentlich	oft	immer
Ich habe wenig Energie.	nie	selten	gelegentlich	oft	immer
Ich habe Muskelverspannungen im Nacken- und Schulterbereich.	nie	selten	gelegentlich	oft	immer
Ich habe Kieferschmerzen.	nie	selten	gelegentlich	oft	immer
Mir wird von manchen Gerüchen, wie z.B. Parfüm, schwindlig und übel.	nie	selten	gelegentlich	oft	immer
Ich muss häufig Wasserlassen.	nie	selten	gelegentlich	oft	immer
Meine Beine fühlen sich unangenehm und ruhelos an, wenn ich versuche nachts einzuschlafen.	nie	selten	gelegentlich	oft	immer
Ich habe Schwierigkeiten, mich an Dinge zu erinnern.	nie	selten	gelegentlich	oft	immer
Ich erlitt als Kind traumatische Erlebnisse.	nie	selten	gelegentlich	oft	immer
Ich habe Schmerzen im Beckenbereich.	nie	selten	gelegentlich	oft	immer

Seite 12

EM01

30. Wie oft während der vergangenen 4 Wochen haben Sie wegen Ihrer Endometriose...

aufgrund von Schmerzen nicht zu geselligen Veranstaltungen gehen können?	Nie	Selten	Manchmal	Häufig	Immer
aufgrund von Schmerzen keine Arbeiten im Haus erledigen können?	Nie	Selten	Manchmal	Häufig	Immer
aufgrund von Schmerzen nur unter Schwierigkeiten stehen können?	Nie	Selten	Manchmal	Häufig	Immer
aufgrund von Schmerzen nur unter Schwierigkeiten sitzen können?	Nie	Selten	Manchmal	Häufig	Immer
aufgrund von Schmerzen nur unter Schwierigkeiten gehen können?	Nie	Selten	Manchmal	Häufig	Immer
aufgrund von Schmerzen nur unter Schwierigkeiten Sport treiben oder den Freizeitbeschäftigungen nachgehen können, denen Sie nachgehen möchten?	Nie	Selten	Manchmal	Häufig	Immer
aufgrund von Schmerzen den Appetit verloren und/oder nicht essen können?	Nie	Selten	Manchmal	Häufig	Immer
aufgrund von Schmerzen nicht gut schlafen können?	Nie	Selten	Manchmal	Häufig	Immer
aufgrund von Schmerzen ins Bett gehen/sich hinlegen müssen?	Nie	Selten	Manchmal	Häufig	Immer
aufgrund von Schmerzen nicht die Dinge tun können die Sie tun wollten?	Nie	Selten	Manchmal	Häufig	Immer
sich nicht in der Lage gefühlt, mit dem Schmerzen umzugehen?	Nie	Selten	Manchmal	Häufig	Immer
sich allgemein Unwohl gefühlt?	Nie	Selten	Manchmal	Häufig	Immer
sich frustriert gefühlt, weil die Symptome nicht besser werden?	Nie	Selten	Manchmal	Häufig	Immer
sich frustriert gefühlt, weil Sie Ihre Symptome nicht kontrollieren können?	Nie	Selten	Manchmal	Häufig	Immer
sich nicht in der Lage gefühlt, die Symptome zu vergessen?	Nie	Selten	Manchmal	Häufig	Immer
das Gefühl gehabt, dass Ihre Symptome Ihr Leben bestimmen?	Nie	Selten	Manchmal	Häufig	Immer
das Gefühl gehabt, dass Ihre Symptome Ihnen Ihr Leben wegnehmen?	Nie	Selten	Manchmal	Häufig	Immer
sich niedergeschlagen gefühlt?	Nie	Selten	Manchmal	Häufig	Immer
sich weinerlich/den Tränen nahe gefühlt?	Nie	Selten	Manchmal	Häufig	Immer
sich elend gefühlt?	Nie	Selten	Manchmal	Häufig	Immer
an Stimmungsschwankungen gelitten?	Nie	Selten	Manchmal	Häufig	Immer
sich schlecht gelaunt oder reizbar gefühlt?	Nie	Selten	Manchmal	Häufig	Immer
sich angriffslustig oder aggressiv gefühlt?	Nie	Selten	Manchmal	Häufig	Immer
Sie sich nicht in der Lage gefühlt, anderen zu erklären, wie Sie sich fühlen?	Nie	Selten	Manchmal	Häufig	Immer
das Gefühl gehabt, dass andere nicht verstehen, was Sie durchleiden?	Nie	Selten	Manchmal	Häufig	Immer

das Gefühl gehabt, dass andere glauben, Sie jammern?	Nie	Selten	Manchmal	Häufig	Immer
sich alleine Gefühlt?	Nie	Selten	Manchmal	Häufig	Immer
sich frustriert gefühlt, weil Sie nicht immer die Kleidung tragen können, die Sie gerne auswählen würden?	Nie	Selten	Manchmal	Häufig	Immer
das Gefühl gehabt, dass Ihr Aussehen beeinflusst wurde?	Nie	Selten	Manchmal	Häufig	Immer
weniger Selbstbewusstsein gehabt?	Nie	Selten	Manchmal	Häufig	Immer

31. Wie oft während der vergangenen 4 Wochen haben Sie wegen Ihrer Endometriose... EM02

auf der Arbeit eine Auszeit nehmen müssen?	Nie	Selten	Manchmal	Häufig	Immer
Ihren Aufgaben am Arbeitsplatz nicht nachkommen können?	Nie	Selten	Manchmal	Häufig	Immer
sich wegen der Symptome auf der Arbeit beschämt/schuldig gefühlt?	Nie	Selten	Manchmal	Häufig	Immer
schuldig gefühlt, weil Sie sich eine Auszeit von der Arbeit genommen haben?	Nie	Selten	Manchmal	Häufig	Immer
Angst gehabt, Ihren Job nicht erledigen zu können?	Nie	Selten	Manchmal	Häufig	Immer

32. Wie oft während der vergangenen 4 Wochen haben Sie wegen Ihrer Endometriose... EM03

Schmerzen während oder nach dem Geschlechtsverkehr gehabt?	Nie	Selten	Manchmal	Häufig	Immer
Sorgen gehabt, Geschlechtsverkehr zu haben?	Nie	Selten	Manchmal	Häufig	Immer
Geschlechtsverkehr wegen der Schmerzen vermieden?	Nie	Selten	Manchmal	Häufig	Immer
sich schuldig gefühlt, weil Sie keinen Geschlechtsverkehr haben wollten?	Nie	Selten	Manchmal	Häufig	Immer
sich frustriert gefühlt, weil Sie den Geschlechtsverkehr nicht genießen können?	Nie	Selten	Manchmal	Häufig	Immer

33. Wie oft fühlten Sie sich im Verlauf der letzten 4 Wochen durch die folgenden Beschwerden beeinträchtigt? GA01

	Überhaupt nicht	An einzelnen Tagen	An mehr als der Hälfte der Tage	Beinahe jeden Tag
Nervosität, Ängstlichkeit oder Anspannung	○	○	○	○
Nicht in der Lage sein, Sorgen zu stoppen oder zu kontrollieren	○	○	○	○
Übermäßige Sorgen bezüglich verschiedener Angelegenheiten	○	○	○	○
Schwierigkeiten zu entspannen	○	○	○	○
Rastlosigkeit, sodass Stillsitzen schwer fällt	○	○	○	○
Schnelle Verärgerung oder Gereiztheit	○	○	○	○
Gefühl der Angst, so als würde etwas Schlimmes passieren	○	○	○	○

34. Bitte geben Sie im folgenden an, wie stark Sie durch Ihre Schmerzen in den verschiedenen Bereichen Ihres Lebens beeinträchtigt sind. Das heißt: Wie sehr hindern die Schmerzen Sie daran, ein normales Leben zu führen? Kreuzen Sie bitte für jeden der sieben Lebensbereiche die Zahl an, die für Sie typische Stärke der Behinderung durch Ihre Schmerzen beschreibt. Ein Wert von 0 bedeutet dabei überhaupt keine Behinderung, und ein Wert von 10 gibt an, dass Sie in diesem Bereich durch die Schmerzen völlig beeinträchtigt sind. PD02

		0	1	2	3	4	5	6	7	8	9	10	
**Familiäre und häusliche Verpflichtungen**<br>(dieser Bereich bezieht sich auf Tätigkeiten, die das Zuhause oder die Familie betreffen. Er umfaßt Hausarbeit und Tätigkeiten rund um das Haus bzw. die Wohnung, auch Gartenarbeiten).	keine Beeinträchtigung	○	○	○	○	○	○	○	○	○	○	○	völlige Beeinträchtigung
**Erholung**<br>(dieser Bereich umfasst Hobbies, Sport und Freizeitaktivitäten)	keine Beeinträchtigung	○	○	○	○	○	○	○	○	○	○	○	völlige Beeinträchtigung
**Soziale Aktivitäten**<br>(dieser Bereich bezieht sich auf das Zusammensein mit Freunden und Bekannten, wie z.B. Feste, Theater – und Konzertbesuche, Essen gehen und andere soziale Aktivitäten)	keine Beeinträchtigung	○	○	○	○	○	○	○	○	○	○	○	völlige Beeinträchtigung
**Beruf**<br>(dieser Bereich bezieht sich auf Aktivitäten, die ein Teil des Berufs sind oder unmittelbar mit dem Beruf zu tun haben; gemeint sind auch Tätigkeiten im Haushalt)	keine Beeinträchtigung	○	○	○	○	○	○	○	○	○	○	○	völlige Beeinträchtigung
**Sexualleben**<br>(dieser Bereich bezieht sich auf die Häufigkeit und die Qualität des Sexuallebens)	keine Beeinträchtigung	○	○	○	○	○	○	○	○	○	○	○	völlige Beeinträchtigung
**Selbstversorgung**<br>(dieser Bereich umfaßt Aktivitäten, die Selbständigkeit und Unabhängigkeit im Alltag ermöglichen, wie z.B. sich waschen und anziehen, Autofahren, ohne dabei auf fremde Hilfe angewiesen zu sein)	keine Beeinträchtigung	○	○	○	○	○	○	○	○	○	○	○	völlige Beeinträchtigung
**Lebensnotwendige Tätigkeiten**<br>(dieser Bereich bezieht sich auf absolut lebensnotwendige Tätigkeiten wie Essen, Schlafen und Atmen)	keine Beeinträchtigung	○	○	○	○	○	○	○	○	○	○	○	völlige Beeinträchtigung

Letzte Seite

Vielen Dank für Ihre Teilnahme!

Wir möchten uns ganz herzlich für Ihre Mithilfe bedanken.

Für Fragen stehen wir Ihnen jederzeit zur Verfügung (janosch-willem.kratz@charite.de)

Ihre Antworten wurden gespeichert, Sie können das Browser-Fenster nun schließen.

Janosch Kratz – 2023
