# Supplementary material for: Evaluating the impact of cannabinoid extracts on chronic pain and quality of life in endometriosis patients – a pilot study
Source: PLoS One. 2026 Sep 22;21(9):e0356768. doi: 10.1371/journal.pone.0356768 (PMC13596799; doi:10.1371/journal.pone.0356768)
Supplement: Supplementary file 3 [file pone.0356768.s002.zip › Monthly (ENDOCAN) en-US.pdf]

Subscribe to DeepL Pro to translate larger documents.
Visit www.DeepL.com/pro for more information.

ENDOCAN → Monthly
12:54 p.m.

September 3, 2024,

AI05

FOLLOW-UP QUESTIONNAIRE for the ENDOCAN Study

Dear Patient,

Your doctor has asked you if you would like to participate in an observational study on the improvement of endometriosis-related symptoms through the use of cannabis extracts. To gain important new insights into the treatment of endometriosis and thereby improve the situation for those affected, we ask that you answer the following questionnaires carefully, on time (before the start of treatment, and after 1, 2, and 3 months of treatment), and truthfully. Please take your time when answering the questions. We are available to answer any questions you may have at any time (janosch-willem.kratz@charite.de).

AI01

1. Please provide us with the following information:

First Name:

Last name:

Date of birth:

Email address:

AI02

2. How much cannabis extract do you currently take in the MORNING?

[Please select]

AI03

3. How much cannabis extract do you currently take in the EVENING?

[Please select]

AI04

4. Did taking the cannabis extract in the past month cause one or more of the following side effects?

Please indicate the intensity of the side effects you experienced that were attributable to taking the cannabis extract.

Fatigue/drowsiness	Not experienced	Mild	moderate	severe
Dry mouth	not experienced	Mild	moderate	severe
Red eyes	not experienced	Mild	moderate	severe
Dizziness	not experienced	Mild	moderate	severe
Nausea	not experienced	Mild	moderate	severe
Impaired motor function/dyscoordination/balance disorders	not experienced	slightly	medium	hard
Cognitive/euphoric effects (“high”)	not felt	Mild	moderate	severe
Difficulty concentrating	not experienced	Mild	moderate	severe
Memory problems	not experienced	Mild	moderate	severe
Anxiety/Panic	not experienced	Mild	moderate	severe
Restlessness/feeling of tension	not felt	Mild	moderate	severe
Paranoia	not experienced	mild	moderate	severe
Heart palpitations > 15 min	not felt	Mild	moderate	severe
Increased appetite	not experienced	slight	moderate	severe
other	not felt	slightly	moderate	severe

AI06

5. Have you experienced any other side effects not listed above?

SA01

6. What was the average intensity of endometriosis-related pain over the past month while on cannabinoid treatment?

- ◯ 0 (no pain)
- ◯ 1
- ◯ 2
- ◯ 3
- ◯ 4
- ◯ 5
- ◯ 6
- ◯ 7
- ◯ 8
- ◯ 9
- ◯ 10 (most severe pain imaginable)

SA02

7. On average, how many days last month did you experience pain due to your endometriosis (despite taking the cannabis extract)?

◯ Last month, I experienced pain on an average of [ ] days ◯ I had pain every day

◯ I had no pain at all

SA04

8. On how many days last month did you take other pain relievers (in addition to the cannabis extract) to relieve your pain?

◯ I took pain relievers on about [ ] days last month ◯ I did not take any pain relievers last month

SA03

9. Do you feel that, because you're taking cannabis extract, you have to rely on other pain relievers?

◯ Yes, significantly less often (about 50% less often) ◯ Yes, somewhat less often (about 25% less often) ◯ No noticeable change

◯ No, I feel like I've had to rely on other pain relievers more often

SA05

10. How often (compared to the previous month) have you used one or more of the following medications to treat your endometriosis-related pain?

Diclofenac (e.g., Voltaren© tablets)	Not currently taking any	Took it less often than last month	Intake unchanged	Taken more often than last month
Buscopan Plus (tablet, coated tablet)	Not currently taking	Taken less frequently than last month	Intake unchanged	Taken more often than last month
Buscopan Plus (suppositories)	Not currently taking	Taken less frequently than last month	Intake unchanged	Taken more often than last month
Acetylsalicylic acid/ASA (e.g., Aspirin©)	Not currently taking any	Taken less frequently than last month	Intake unchanged	Taken more often than last month
Celecoxib (e.g., Celebrex©)	Not currently taking	Taken less frequently than last month	Intake unchanged	Taken more often than last month
Ibuprofen	Not currently taking it	Taken less often than last month	Intake unchanged	Taken more often than last month
Acetaminophen	Not currently taking any	Taken less often than last month	Intake unchanged	Taken more often than last month
Metamizole (Novaminsulfon©)	Not currently taking	Taken less frequently than last month	Intake unchanged	Taken more often than last month
Tramadol (e.g., Tramal©)	Not currently taking	Taken less frequently than last month	Intake unchanged	Taken more often than last month
Tilidine	Not currently taking	Taken less often than last month	Intake unchanged	Taken more often than last month
Dihydrocodeine	Not currently taking	Taken less often than last month	Intake unchanged	Taken more often than last month
Morphine	Not currently taking any	Taken less frequently than last month	Intake unchanged	Taken more often than last month
Oxycodone	Not currently taking any	Taken less often than last month	Intake unchanged	Taken more often than last month
Levomethadone	Not currently taking it	Taken less frequently than last month	Intake unchanged	Taken more often than last month

Fentanyl	Not currently taking	Taken less often than last month	Intake unchanged	Taken more often than last month
Pethidine	Not currently taking	Taken less often than last month	Intake unchanged	Taken more often than last month
Buprenorphine	Not currently taking	Taken less often than last month	Intake unchanged	Taken more often than last month
Piritramide	Not currently taking	Taken less often than last month	Intake unchanged	Taken more often than last month
Co-analgesics such as amitriptyline, doxepin, clomipramine, imipramine	Not currently taking any	Taken less frequently than last month	Intake unchanged	Taken more often than last month
Co-analgesics such as carbamazepine, gabapentin, pregabalin	Not currently taking any	Taken less frequently than last month	Intake unchanged	Taken more often than last month
Benzodiazepines (diazepam, lorazepam, lormetazepam, midazolam, oxazepam, temazepam, flunitrazepam)	Not currently taking any	Taken less frequently than last month	same frequency of use	Taken more often than last month

11. Have you taken any other pain relievers not listed above? SA06

CF01

12. We'd like to learn more about the problems you've had in the past month because you've felt tired, weak, or lacking energy. Please answer ALL questions by checking the answer that best applies to you. If you've been feeling tired for a long time, compare how you feel now to how you felt the last time you felt well.

Please check only one box per line.

Do you have problems with fatigue?	less than usual	no more than usual (unchanged)	more than usual	much more than usual
Do you need to rest more often?	less than usual	no more than usual (unchanged)	more than usual	much more than usual
Do you feel tired or sleepy?	less than usual	no more than usual (unchanged)	more than usual	much more than usual
Do you find it hard to get started on tasks?	less than usual	no more than usual (unchanged)	more than usual	much more than usual
Do you feel weak or lacking in energy?	less than usual	no more than usual (unchanged)	more than usual	much more than usual
Do you have less strength in your muscles?	less than usual	no more than usual (unchanged)	more than usual	much more than usual
Do you feel weak?	less than usual	no more than usual (unchanged)	more than usual	much more than usual
Do you have trouble concentrating?	less than usual	no more than usual (unchanged)	more than usual	much more than usual
Do you frequently slip up when speaking?	less than usual	no more than usual (unchanged)	more than usual	much more than usual
Do you find it harder to find the right word?	less than usual	no more than usual (unchanged)	more than usual	much more than usual

CF02

13. Rate your memory performance

How would you rate your memory?	Better than usual	as usual	worse than usual	much worse than usual

CS01

14. For each statement, please select the answer on the right that best applies to you at this time.

When I wake up in the morning, I feel tired and not well-rested.	never	Rarely	occasionally	often	always
My muscles feel stiff and sore.	never	Rarely	occasionally	often	always
I have anxiety attacks.	never	Rarely	occasionally	often	always
I grind or clench my teeth.	never	rarely	occasionally	often	always
I have problems with diarrhea and/or constipation.	never	Rarely	occasionally	often	always
I need help with my daily activities.	never	Rarely	occasionally	often	always
I am sensitive to bright light.	never	Rarely	occasionally	often	always
I get tired very quickly during physical activities.	never	rarely	occasionally	often	always
I have pain all over my body.	never	Rarely	occasionally	often	always
I have a headache.	never	rarely	occasionally	often	always
My bladder feels uncomfortable and/or I have a burning sensation when I urinate.	never	Rarely	occasionally	often	always
I don't sleep well.	never	rarely	occasionally	often	always
I have trouble concentrating.	never	rarely	occasionally	often	always
I have skin problems, such as dry or itchy skin or a rash.	never	Rarely	occasionally	often	always
Stress makes my physical symptoms worse.	never	Rarely	occasionally	often	always
I feel sad or down.	never	Rarely	occasionally	often	always
I have little energy.	never	Rarely	occasionally	often	always
I have muscle tension in my neck and shoulders.	never	Rarely	occasionally	often	always
I have a toothache.	never	rarely	occasionally	often	always
Certain smells, such as perfume, make me feel dizzy and nauseous.	never	Rarely	occasionally	often	always
I have to urinate frequently.	never	Rarely	occasionally	often	always
My legs feel uncomfortable and restless when I try to fall asleep at night.	never	Rarely	occasionally	often	always
I have trouble remembering things.	never	rarely	occasionally	often	always
I experienced traumatic events as a child.	never	Rarely	occasionally	often	always
I have pain in my pelvic area.	never	Rarely	occasionally	often	always

EM01

15. How often during the past 4 weeks have you, because of your endometriosis...

been unable to attend social events because of pain?	Never	Rarely	Sometimes	Often	Always
unable to do any housework because of pain?	Never	Rarely	Sometimes	Often	Always
Do you have difficulty standing due to pain?	Never	Rarely	Sometimes	Often	Always
Do you have difficulty sitting because of pain?	Never	Rarely	Sometimes	Often	Always
Do you have difficulty walking due to pain?	Never	Rarely	Sometimes	Often	Always
Do you find it difficult to exercise or engage in the leisure activities you'd like to do because of pain?	Never	Rarely	Sometimes	Often	Always
Have you lost your appetite and/or been unable to eat because of pain?	Never	Rarely	Sometimes	Often	Always
unable to sleep well because of pain?	Never	Rarely	Sometimes	Often	Always
Do you have to go to bed or lie down because of pain?	Never	Rarely	Sometimes	Often	Always
unable to do the things you wanted to do because of pain?	Never	Rarely	Sometimes	Often	Always
felt unable to cope with the pain?	Never	Rarely	Sometimes	Often	Always
Do you generally feel unwell?	Never	Rarely	Sometimes	Often	Always
felt frustrated because your symptoms aren't getting better?	Never	Rarely	Sometimes	Often	Always
felt frustrated because you can't control your symptoms?	Never	Rarely	Sometimes	Often	Always
Did you feel unable to forget the symptoms?	Never	Rarely	Sometimes	Often	Always
felt that your symptoms are controlling your life?	Never	Rarely	Sometimes	Often	Always
felt like your symptoms were taking your life away?	Never	Rarely	Sometimes	Often	Always
felt down?	Never	Rarely	Sometimes	Often	Always
felt tearful or close to tears?	Never	Rarely	Sometimes	Often	Always
felt miserable?	Never	Rarely	Sometimes	Often	Always
Have you ever suffered from mood swings?	Never	Rarely	Sometimes	Often	Always
felt in a bad mood or irritable?	Never	Rarely	Sometimes	Often	Always
Have you ever felt hostile or aggressive?	Never	Rarely	Sometimes	Often	Always
Have you ever felt unable to explain to others how you feel?	Never	Rarely	Sometimes	Often	Always
felt that others don't understand what you're going through?	Never	Rarely	Sometimes	Often	Always

felt that others think you're complaining?	Never	Rarely	Sometimes	Often	Always
Alone?	Never	Rarely	Sometimes	Often	Always
felt frustrated because you can't always wear the clothes you'd like to choose?	Never	Rarely	Sometimes	Often	Always
felt that your appearance was affected?	Never	Rarely	Sometimes	Often	Always
had less self-confidence?	Never	Rarely	Sometimes	Often	Always

16. How often during the past 4 weeks have you, because of your endometriosis...

EM02

had to take time off work?	Never	Rarely	Sometimes	Often	Always
unable to perform your job duties?	Never	Rarely	Sometimes	Often	Always
felt ashamed or guilty at work because of your symptoms?	Never	Rarely	Sometimes	Often	Always
Do you feel guilty because you took time off from work?	Never	Rarely	Sometimes	Often	Always
Have you ever been afraid that you wouldn't be able to do your job?	Never	Rarely	Sometimes	Often	Always

17. How often during the past 4 weeks have you, because of your endometriosis...

EM03

experienced pain during or after sexual intercourse?	Never	Rarely	Sometimes	Often	Always
Have you ever been worried about having sexual intercourse?	Never	Rarely	Sometimes	Often	Always
Have you avoided sexual intercourse because of the pain?	Never	Rare	Sometimes	Often	Always
felt guilty because you didn't want to have sexual intercourse?	Never	Rarely	Sometimes	Often	Always
felt frustrated because you can't enjoy sexual intercourse?	Never	Rarely	Sometimes	Often	Always

GA01

18. How often have you felt impaired by the following symptoms over the past 4 weeks?

	Not at all	On some days	On more than half the days	Almost every day

Nervousness, anxiety, or tension

Being unable to stop or control worries Excessive worry about

various matters Difficulty relaxing

Restlessness, making it hard to sit still

Getting annoyed or irritated easily

A feeling of fear, as if something bad were going to happen

PD02

19. Please indicate below to what extent your pain affects you in the various areas of your life. In other words: To what extent does the pain prevent you from leading a normal life? For each of the seven areas of life, please check the number that best describes the typical severity of the impairment caused by your pain. A score of 0 means no impairment at all, and a score of 10 indicates that you are completely impaired in that area due to the pain.

Family and household responsibilities

(This category refers to activities related to the home or the family. It includes housework and activities around the house or apartment, including yard work).

	0	1	2	3	4	5	6	7	8	9	10	
no impairment	○	○	○	○	○	○	○	○	○	○	○	Complete impairment

Recreation

(This category includes hobbies, sports, and leisure activities)

	0	1	2	3	4	5	6	7	8	9	10	
none Impairment	○	○	○	○	○	○	○	○	○	○	○	All Impairment

Social activities

(This section refers to spending time with friends and acquaintances, such as parties, going to the theater or concerts, dining out, and other social activities)

	0	1	2	3	4	5	6	7	8	9	10	
no impairment	○	○	○	○	○	○	○	○	○	○	○	Complete impairment

Work

(This area refers to Activities that are part of one's occupation or directly related to the occupation; this also includes household activities)

	0	1	2	3	4	5	6	7	8	9	10	
none impairment	○	○	○	○	○	○	○	○	○	○	○	Compl ete impairment

Sexual life

(this area refers to

Frequency and quality of sexual life)

	0	1	2	3	4	5	6	7	8	9	10	
none Impairment	○	○	○	○	○	○	○	○	○	○	○	entirel y Impairment
	○	○	○	○	○	○	○	○	○	○	○	

Self-sufficiency

(This area encompasses activities that enable autonomy and independence in daily life, such as washing and dressing oneself, and driving a car, without having to rely on outside help)

	0	1	2	3	4	5	6	7	8	9	10	
no impairment	○	○	○	○	○	○	○	○	○	○	○	Complete impairment

Activities Essential for Daily Living

(This category refers to absolutely (vital activities such as eating, sleeping, and breathing)

	0	1	2	3	4	5	6	7	8	9	10	
no Impairment	○	○	○	○	○	○	○	○	○	○	○	Compl ete impairment

Thank you very much for your participation!

We would like to express our sincere gratitude for your assistance.

Your answers have been saved; you can now close the browser window.

Janosch Kratz – 2023
