# Supplementary material for: Evaluating the impact of cannabinoid extracts on chronic pain and quality of life in endometriosis patients – a pilot study
Source: PLoS One. 2026 Sep 22;21(9):e0356768. doi: 10.1371/journal.pone.0356768 (PMC13596799; doi:10.1371/journal.pone.0356768)
Supplement: Supplementary file 3 [file pone.0356768.s002.zip › Monthly (ENDOCAN).pdf]

ENDOCAN → Monthly 03.09.2024, 12:54

Seite 01
Info

AI05

FRAGEBOGEN ZUR VERLAUFSKONTROLLE der ENDOCAN-Studie

Sehr geehrte Patientin,

Sie sind von Ihrem Arzt/Ihrer Ärztin gefragt worden, ob Sie an einer Beobachtungsstudie bezüglich der Verbesserung von endometriosebedingten Symptomen durch die Einnahme von Cannabisextrakten teilnehmen möchten. Um wichtige neue Erkenntnisse bezüglich der Behandlung von Endometriose zu erforschen und somit die Situation für die betroffenen Personen verbessern zu können bitten wir Sie die folgenden Fragebögen sorgfältig, pünktlich (vor Behandlungsbeginn, nach 1,2 und 3 Monaten Behandlung) und wahrheitsgemäß zu beantworten. Bitte nehmen Sie sich für die Beantwortung ausreichend Zeit. Für Fragen stehen wir Ihnen jederzeit zur Verfügung (janosch-willem.kratz@charite.de).

AI01

1. Bitte teilen Sie uns die folgenden Informationen mit:

Vorname:

Nachname:

Geburtsdatum:

Emailadresse:

AI02

2. Welche Menge des Cannabisextraktes nehmen Sie momentan MORGENS ein?

[Bitte auswählen]

AI03

3. Welche Menge des Cannabisextraktes nehmen Sie momentan ABENDS ein?

[Bitte auswählen]

AI04

4. Hat die Einnahme des Cannabisextraktes im letzten Monat zu einer oder mehreren der folgenden Nebenwirkungen geführt?

Bitte geben Sie die Intensität der wahrgenommenen Nebenwirkungen an, die auf die Einnahme des Cannabisextraktes zurückzuführen sind.

Müdigkeit/Schläfrigkeit	nicht verspürt	leicht	mittel	stark
Mundtrockenheit	nicht verspürt	leicht	mittel	stark
Gerötete Augen	nicht verspürt	leicht	mittel	stark
Schwindel	nicht verspürt	leicht	mittel	stark
Übelkeit	nicht verspürt	leicht	mittel	stark
Eingeschränkte Motorik/Dyskoordination/Gleichgewichtsstörungen	nicht verspürt	leicht	mittel	stark
Kognitive/berauschende Wirkung („High“-Gefühl)	nicht verspürt	leicht	mittel	stark
Konzentrationsschwierigkeiten	nicht verspürt	leicht	mittel	stark
Gedächtnisstörungen	nicht verspürt	leicht	mittel	stark
Angst/Panik	nicht verspürt	leicht	mittel	stark
Unruhe/Spannungsgefühl	nicht verspürt	leicht	mittel	stark
Paranoia	nicht verspürt	leicht	mittel	stark
Herzrasen > 15Min	nicht verspürt	leicht	mittel	stark
Appetitsteigerung	nicht verspürt	leicht	mittel	stark
andere	nicht verspürt	leicht	mittel	stark

AI06

5. Haben sie weitere Nebenwirkungen verspürt, die in der vorherigen Liste nicht aufgeführt sind?

Seite 02

Pain

SA01

6. Wie war die durchschnittliche endometriosebedingte Schmerzstärke im letzten Monat unter der Cannabinoidbehandlung?

- ◯ 0 (kein Schmerz)
- ◯ 1
- ◯ 2
- ◯ 3
- ◯ 4
- ◯ 5
- ◯ 6
- ◯ 7
- ◯ 8
- ◯ 9
- ◯ 10 (stärkster vorstellbarer Schmerz)

SA02

7. An wie vielen Tagen hatten Sie im letzten Monat durchschnittlich Schmerzen aufgrund Ihrer Endometriose (trotz Einnahme des Cannbisextraktes) ?

- ◯ Ich hatte im letzten Monat an durchschnittlich [ ] Tagen Schmerzen
- ◯ Ich hatte jeden Tag Schmerzen
- ◯ Ich hatte keinerlei Schmerzen

SA04

8. An wie vielen Tagen haben Sie im letzten Monat andere Schmerzmittel (zusätzlich zum Cannbisextrakt) eingenommen um ihre Schmerzen zu lindern?

- ◯ Ich habe letzten Monat an ca. [ ] Tagen Schmerzmittel eingenommen
- ◯ Ich habe im letzten Monat keine Schmerzmittel eingenommen

SA03

9. Haben Sie den Eindruck, dass Sie aufgrund der Einnahme des Cannabisextraktes seltener zu anderen Schmerzmitteln greifen müssen?

- ◯ Ja, deutlich seltener (ca. 50% seltener)
- ◯ Ja, etwas seltener (ca. 25% seltener)
- ◯ Keine Veränderung feststellbar
- ◯ Nein, ich habe den Eindruck ich musste häufiger zu anderen Schmerzmitteln greifen

SA05

10. Wie häufig haben Sie (im Vergleich zum vorherigen Monat) eines/mehrere der folgenden Medikamente verwendet,um ihre endometriosebedingten Schmerzen zu behandeln?

Diclofenac (z.B. Voltaren© Tabletten)	Momentan keine Einnahme	Seltener eingenommen als im letzten Monat	unveränderte Einnahme	Häufiger eingenommen als im letzten Monat
Buscopan Plus (Tablette, Dragee)	Momentan keine Einnahme	Seltener eingenommen als im letzten Monat	unveränderte Einnahme	Häufiger eingenommen als im letzten Monat
Buscopan Plus (Zäpfchen)	Momentan keine Einnahme	Seltener eingenommen als im letzten Monat	unveränderte Einnahme	Häufiger eingenommen als im letzten Monat
Acetylsalicylsäure/ASS (z.B. Aspirin©)	Momentan keine Einnahme	Seltener eingenommen als im letzten Monat	unveränderte Einnahme	Häufiger eingenommen als im letzten Monat
Celecoxib (z.B. Celebrex©)	Momentan keine Einnahme	Seltener eingenommen als im letzten Monat	unveränderte Einnahme	Häufiger eingenommen als im letzten Monat
Ibuprofen	Momentan keine Einnahme	Seltener eingenommen als im letzten Monat	unveränderte Einnahme	Häufiger eingenommen als im letzten Monat
Paracetamol	Momentan keine Einnahme	Seltener eingenommen als im letzten Monat	unveränderte Einnahme	Häufiger eingenommen als im letzten Monat
Metamizol (Novaminsulfon©)	Momentan keine Einnahme	Seltener eingenommen als im letzten Monat	unveränderte Einnahme	Häufiger eingenommen als im letzten Monat
Tramadol (z.B. Tramal©)	Momentan keine Einnahme	Seltener eingenommen als im letzten Monat	unveränderte Einnahme	Häufiger eingenommen als im letzten Monat
Tilidin	Momentan keine Einnahme	Seltener eingenommen als im letzten Monat	unveränderte Einnahme	Häufiger eingenommen als im letzten Monat
Dihydrocodein	Momentan keine Einnahme	Seltener eingenommen als im letzten Monat	unveränderte Einnahme	Häufiger eingenommen als im letzten Monat
Morphin	Momentan keine Einnahme	Seltener eingenommen als im letzten Monat	unveränderte Einnahme	Häufiger eingenommen als im letzten Monat
Oxycodon	Momentan keine Einnahme	Seltener eingenommen als im letzten Monat	unveränderte Einnahme	Häufiger eingenommen als im letzten Monat
Levomethadon	Momentan keine Einnahme	Seltener eingenommen als im letzten Monat	unveränderte Einnahme	Häufiger eingenommen als im letzten Monat

Fentanyl	Momentan keine Einnahme	Seltener eingenommen als im letzten Monat	unveränderte Einnahme	Häufiger eingenommen als im letzten Monat
Pethidin	Momentan keine Einnahme	Seltener eingenommen als im letzten Monat	unveränderte Einnahme	Häufiger eingenommen als im letzten Monat
Buprenorphin	Momentan keine Einnahme	Seltener eingenommen als im letzten Monat	unveränderte Einnahme	Häufiger eingenommen als im letzten Monat
Piritramid	Momentan keine Einnahme	Seltener eingenommen als im letzten Monat	unveränderte Einnahme	Häufiger eingenommen als im letzten Monat
Koanalgetika wie Amitriptylin, Doxepin, Clomipramin, Imipramin	Momentan keine Einnahme	Seltener eingenommen als im letzten Monat	unveränderte Einnahme	Häufiger eingenommen als im letzten Monat
Ko-Analgetika wie Carbamazepin, Gabapentin, Pregabalin	Momentan keine Einnahme	Seltener eingenommen als im letzten Monat	unveränderte Einnahme	Häufiger eingenommen als im letzten Monat
Benzodiazepine (Diazepam, Lorazepam, Lormetazepam, Midazolam, Oxazepam, Temazepam, Flunitrazepam)	Momentan keine Einnahme	Seltener eingenommen als im letzten Monat	unveränderte Einnahme	Häufiger eingenommen als im letzten Monat

11. Haben sie weitere Schmerzmittel eingenommen die in der vorherigen Liste nicht aufgeführt sind? SA06

CF01

12. Wir würden gerne mehr darüber erfahren, welche Probleme Sie im letzten Monat hatten, weil Sie sich müde, schwach oder energielos fühlten. Bitte beantworten Sie ALLE Fragen, indem Sie die Antwort ankreuzen, die am ehesten auf Sie zutrifft. Wenn Sie sich schon lange müde fühlen, vergleichen Sie sich mit dem Gefühl, als es Ihnen zuletzt gut ging.

Bitte kreuzen Sie nur ein Kästchen pro Zeile an.

Haben Sie Probleme mit Müdigkeit?	weniger als sonst	nicht mehr als üblich (gleichbleibend)	mehr als sonst	viel mehr als sonst
Müssen Sie sich häufiger ausruhen?	weniger als sonst	nicht mehr als üblich (gleichbleibend)	mehr als sonst	viel mehr als sonst
Fühlen Sie sich müde oder schläfrig?	weniger als sonst	nicht mehr als üblich (gleichbleibend)	mehr als sonst	viel mehr als sonst
Fällt es Ihnen schwer, Aufgaben zu beginnen?	weniger als sonst	nicht mehr als üblich (gleichbleibend)	mehr als sonst	viel mehr als sonst
Fühlen Sie sich kraftlos/ohne Energie?	weniger als sonst	nicht mehr als üblich (gleichbleibend)	mehr als sonst	viel mehr als sonst
Haben Sie weniger Kraft in Ihren Muskeln?	weniger als sonst	nicht mehr als üblich (gleichbleibend)	mehr als sonst	viel mehr als sonst
Fühlen Sie sich schwach?	weniger als sonst	nicht mehr als üblich (gleichbleibend)	mehr als sonst	viel mehr als sonst
Haben Sie Konzentrationsschwierigkeiten?	weniger als sonst	nicht mehr als üblich (gleichbleibend)	mehr als sonst	viel mehr als sonst
Machen Sie beim Reden häufig Versprecher?	weniger als sonst	nicht mehr als üblich (gleichbleibend)	mehr als sonst	viel mehr als sonst
Fällt es Ihnen schwerer, das richtige Wort zu finden?	weniger als sonst	nicht mehr als üblich (gleichbleibend)	mehr als sonst	viel mehr als sonst

CF02

13. Beurteilen Sie ihre Gedächtnisleistung

Wie würden Sie ihre Gedächtnisleistung beurteilen?	besser als sonst	wie üblich	schlechter als sonst	viel schlechter als sonst

CS01

14. Bitte markieren Sie bei jeder Aussage die für Sie aktuell am besten passende Antwort auf der rechten Seite.

Wenn ich morgens aufwache, fühle ich mich müde und nicht erholt.	nie	selten	gelegentlich	oft	immer
Meine Muskeln fühlen sich steif und schmerzhaft an.	nie	selten	gelegentlich	oft	immer
Ich habe Angstattacken.	nie	selten	gelegentlich	oft	immer
Ich knirsche oder beiße meine Zähne zusammen.	nie	selten	gelegentlich	oft	immer
Ich habe Probleme mit Durchfall und/oder Verstopfung.	nie	selten	gelegentlich	oft	immer
Ich brauche Hilfe bei der Verrichtung meiner Alltagstätigkeiten.	nie	selten	gelegentlich	oft	immer
Ich reagiere empfindlich auf helles Licht.	nie	selten	gelegentlich	oft	immer
Ich ermüde sehr schnell bei körperlichen Aktivitäten.	nie	selten	gelegentlich	oft	immer
Ich habe am ganzen Körper Schmerzen.	nie	selten	gelegentlich	oft	immer
Ich habe Kopfschmerzen.	nie	selten	gelegentlich	oft	immer
Meine Blase fühlt sich unangenehm an und/oder ich habe Brennen beim Wasserlassen.	nie	selten	gelegentlich	oft	immer
Ich schlafe nicht gut.	nie	selten	gelegentlich	oft	immer
Ich habe Konzentrationsschwierigkeiten.	nie	selten	gelegentlich	oft	immer
Ich habe Hautprobleme, wie z.B. trockene oder juckende Haut oder Hautausschlag.	nie	selten	gelegentlich	oft	immer
Stress verstärkt meine körperlichen Beschwerden.	nie	selten	gelegentlich	oft	immer
Ich fühle mich traurig oder niedergeschlagen.	nie	selten	gelegentlich	oft	immer
Ich habe wenig Energie.	nie	selten	gelegentlich	oft	immer
Ich habe Muskelverspannungen im Nacken- und Schulterbereich.	nie	selten	gelegentlich	oft	immer
Ich habe Kieferschmerzen.	nie	selten	gelegentlich	oft	immer
Mir wird von manchen Gerüchen, wie z.B. Parfüm, schwindlig und übel.	nie	selten	gelegentlich	oft	immer
Ich muss häufig Wasserlassen.	nie	selten	gelegentlich	oft	immer
Meine Beine fühlen sich unangenehm und ruhelos an, wenn ich versuche nachts einzuschlafen.	nie	selten	gelegentlich	oft	immer
Ich habe Schwierigkeiten, mich an Dinge zu erinnern.	nie	selten	gelegentlich	oft	immer
Ich erlitt als Kind traumatische Erlebnisse.	nie	selten	gelegentlich	oft	immer
Ich habe Schmerzen im Beckenbereich.	nie	selten	gelegentlich	oft	immer

Seite 05

EHP 30

EM01

15. Wie oft während der vergangenen 4 Wochen haben Sie wegen Ihrer Endometriose...

aufgrund von Schmerzen nicht zu geselligen Veranstaltungen gehen können?	Nie	Selten	Manchmal	Häufig	Immer
aufgrund von Schmerzen keine Arbeiten im Haus erledigen können?	Nie	Selten	Manchmal	Häufig	Immer
aufgrund von Schmerzen nur unter Schwierigkeiten stehen können?	Nie	Selten	Manchmal	Häufig	Immer
aufgrund von Schmerzen nur unter Schwierigkeiten sitzen können?	Nie	Selten	Manchmal	Häufig	Immer
aufgrund von Schmerzen nur unter Schwierigkeiten gehen können?	Nie	Selten	Manchmal	Häufig	Immer
aufgrund von Schmerzen nur unter Schwierigkeiten Sport treiben oder den Freizeitbeschäftigungen nachgehen können, denen Sie nachgehen möchten?	Nie	Selten	Manchmal	Häufig	Immer
aufgrund von Schmerzen den Appetit verloren und/oder nicht essen können?	Nie	Selten	Manchmal	Häufig	Immer
aufgrund von Schmerzen nicht gut schlafen können?	Nie	Selten	Manchmal	Häufig	Immer
aufgrund von Schmerzen ins Bett gehen/sich hinlegen müssen?	Nie	Selten	Manchmal	Häufig	Immer
aufgrund von Schmerzen nicht die Dinge tun können die Sie tun wollten?	Nie	Selten	Manchmal	Häufig	Immer
sich nicht in der Lage gefühlt, mit dem Schmerzen umzugehen?	Nie	Selten	Manchmal	Häufig	Immer
sich allgemein Unwohl gefühlt?	Nie	Selten	Manchmal	Häufig	Immer
sich frustriert gefühlt, weil die Symptome nicht besser werden?	Nie	Selten	Manchmal	Häufig	Immer
sich frustriert gefühlt, weil Sie Ihre Symptome nicht kontrollieren können?	Nie	Selten	Manchmal	Häufig	Immer
sich nicht in der Lage gefühlt, die Symptome zu vergessen?	Nie	Selten	Manchmal	Häufig	Immer
das Gefühl gehabt, dass Ihre Symptome Ihr Leben bestimmen?	Nie	Selten	Manchmal	Häufig	Immer
das Gefühl gehabt, dass Ihre Symptome Ihnen Ihr Leben wegnehmen?	Nie	Selten	Manchmal	Häufig	Immer
sich niedergeschlagen gefühlt?	Nie	Selten	Manchmal	Häufig	Immer
sich weinerlich/den Tränen nahe gefühlt?	Nie	Selten	Manchmal	Häufig	Immer
sich elend gefühlt?	Nie	Selten	Manchmal	Häufig	Immer
an Stimmungsschwankungen gelitten?	Nie	Selten	Manchmal	Häufig	Immer
sich schlecht gelaunt oder reizbar gefühlt?	Nie	Selten	Manchmal	Häufig	Immer
sich angriffslustig oder aggressiv gefühlt?	Nie	Selten	Manchmal	Häufig	Immer
Sie sich nicht in der Lage gefühlt, anderen zu erklären, wie Sie sich fühlen?	Nie	Selten	Manchmal	Häufig	Immer
das Gefühl gehabt, dass andere nicht verstehen, was Sie durchleiden?	Nie	Selten	Manchmal	Häufig	Immer

das Gefühl gehabt, dass andere glauben, Sie jammern?	Nie	Selten	Manchmal	Häufig	Immer
sich alleine Gefühlt?	Nie	Selten	Manchmal	Häufig	Immer
sich frustriert gefühlt, weil Sie nicht immer die Kleidung tragen können, die Sie gerne auswählen würden?	Nie	Selten	Manchmal	Häufig	Immer
das Gefühl gehabt, dass Ihr Aussehen beeinflusst wurde?	Nie	Selten	Manchmal	Häufig	Immer
weniger Selbstbewusstsein gehabt?	Nie	Selten	Manchmal	Häufig	Immer

EM02

16. Wie oft während der vergangenen 4 Wochen haben Sie wegen Ihrer Endometriose...

auf der Arbeit eine Auszeit nehmen müssen?	Nie	Selten	Manchmal	Häufig	Immer
Ihren Aufgaben am Arbeitsplatz nicht nachkommen können?	Nie	Selten	Manchmal	Häufig	Immer
sich wegen der Symptome auf der Arbeit beschämt/schuldig gefühlt?	Nie	Selten	Manchmal	Häufig	Immer
schuldig gefühlt, weil Sie sich eine Auszeit von der Arbeit genommen haben?	Nie	Selten	Manchmal	Häufig	Immer
Angst gehabt, Ihren Job nicht erledigen zu können?	Nie	Selten	Manchmal	Häufig	Immer

EM03

17. Wie oft während der vergangenen 4 Wochen haben Sie wegen Ihrer Endometriose...

Schmerzen während oder nach dem Geschlechtsverkehr gehabt?	Nie	Selten	Manchmal	Häufig	Immer
Sorgen gehabt, Geschlechtsverkehr zu haben?	Nie	Selten	Manchmal	Häufig	Immer
Geschlechtsverkehr wegen der Schmerzen vermieden?	Nie	Selten	Manchmal	Häufig	Immer
sich schuldig gefühlt, weil Sie keinen Geschlechtsverkehr haben wollten?	Nie	Selten	Manchmal	Häufig	Immer
sich frustriert gefühlt, weil Sie den Geschlechtsverkehr nicht genießen können?	Nie	Selten	Manchmal	Häufig	Immer

Seite 06
GAD 7

GA01

18. Wie oft fühlten Sie sich im Verlauf der letzten 4 Wochen durch die folgenden Beschwerden beeinträchtigt?

	Überhaupt nicht	An einzelnen Tagen	An mehr als der Hälfte der Tage	Beinahe jeden Tag
Nervosität, Ängstlichkeit oder Anspannung	○	○	○	○
Nicht in der Lage sein, Sorgen zu stoppen oder zu kontrollieren	○	○	○	○
Übermäßige Sorgen bezüglich verschiedener Angelegenheiten	○	○	○	○
Schwierigkeiten zu entspannen	○	○	○	○
Rastlosigkeit, sodass Stillsitzen schwer fällt	○	○	○	○
Schnelle Verärgerung oder Gereiztheit	○	○	○	○
Gefühl der Angst, so als würde etwas Schlimmes passieren	○	○	○	○

PD02

19. Bitte geben Sie im folgenden an, wie stark Sie durch Ihre Schmerzen in den verschiedenen Bereichen Ihres Lebens beeinträchtigt sind. Das heißt: Wie sehr hindern die Schmerzen Sie daran, ein normales Leben zu führen? Kreuzen Sie bitte für jeden der sieben Lebensbereiche die Zahl an, die für Sie typische Stärke der Behinderung durch Ihre Schmerzen beschreibt. Ein Wert von 0 bedeutet dabei überhaupt keine Behinderung, und ein Wert von 10 gibt an, dass Sie in diesem Bereich durch die Schmerzen völlig beeinträchtigt sind.

		0	1	2	3	4	5	6	7	8	9	10	
**Familiäre und häusliche Verpflichtungen**<br>(dieser Bereich bezieht sich auf Tätigkeiten, die das Zuhause oder die Familie betreffen. Er umfaßt Hausarbeit und Tätigkeiten rund um das Haus bzw. die Wohnung, auch Gartenarbeiten).	keine Beeinträchtigung	○	○	○	○	○	○	○	○	○	○	○	völlige Beeinträchtigung
**Erholung**<br>(dieser Bereich umfasst Hobbies, Sport und Freizeitaktivitäten)	keine Beeinträchtigung	○	○	○	○	○	○	○	○	○	○	○	völlige Beeinträchtigung
**Soziale Aktivitäten**<br>(dieser Bereich bezieht sich auf das Zusammensein mit Freunden und Bekannten, wie z.B. Feste, Theater – und Konzertbesuche, Essen gehen und andere soziale Aktivitäten)	keine Beeinträchtigung	○	○	○	○	○	○	○	○	○	○	○	völlige Beeinträchtigung
**Beruf**<br>(dieser Bereich bezieht sich auf Aktivitäten, die ein Teil des Berufs sind oder unmittelbar mit dem Beruf zu tun haben; gemeint sind auch Tätigkeiten im Haushalt)	keine Beeinträchtigung	○	○	○	○	○	○	○	○	○	○	○	völlige Beeinträchtigung
**Sexualleben**<br>(dieser Bereich bezieht sich auf die Häufigkeit und die Qualität des Sexuallebens)	keine Beeinträchtigung	○	○	○	○	○	○	○	○	○	○	○	völlige Beeinträchtigung
**Selbstversorgung**<br>(dieser Bereich umfaßt Aktivitäten, die Selbständigkeit und Unabhängigkeit im Alltag ermöglichen, wie z.B. sich waschen und anziehen, Autofahren, ohne dabei auf fremde Hilfe angewiesen zu sein)	keine Beeinträchtigung	○	○	○	○	○	○	○	○	○	○	○	völlige Beeinträchtigung
**Lebensnotwendige Tätigkeiten**<br>(dieser Bereich bezieht sich auf absolut lebensnotwendige Tätigkeiten wie Essen, Schlafen und Atmen)	keine Beeinträchtigung	○	○	○	○	○	○	○	○	○	○	○	völlige Beeinträchtigung

Letzte Seite

Vielen Dank für Ihre Teilnahme!

Wir möchten uns ganz herzlich für Ihre Mithilfe bedanken.

Ihre Antworten wurden gespeichert, Sie können das Browser-Fenster nun schließen.

Janosch Kratz – 2023
